# Metabolomics combined with network pharmacology exploration reveals the modulatory properties of *Astragali Radix* extract in the treatment of liver fibrosis

**DOI:** 10.1186/s13020-019-0251-z

**Published:** 2019-08-28

**Authors:** Dan Wang, Ruisheng Li, Shizhang Wei, Sijia Gao, Zhuo Xu, Honghong Liu, Ruilin Wang, Haotian Li, Huadan Cai, Jian Wang, Yanling Zhao

**Affiliations:** 1 Provincial and State Key Laboratory Breeding Base of System Research, Development and Utilization of Chinese Herbal Medicine Resources, College of Pharmacy, Chengdu University of Traditional Chinese Medicine, Chengdu, 611137 China; 2 Department of Pharmacy, The Fifth Medical Center of PLA General Hospital, Beijing, 100039 China; 3 Research Center for Clinical and Translational Medicine, The Fifth Medical Center of PLA General Hospital, Beijing, 100039 China; 4 Integrative Medical Center, The Fifth Medical Center of PLA General Hospital, Beijing, 100039 China; 5 Department of Traditional Chinese Medicine, The Fifth Medical Center of PLA General Hospital, Beijing, 100039 China

**Keywords:** *Astragali Radix*, Liver fibrosis, Metabolomics, Network pharmacology, Modulatory properties, Molecular mechanisms

## Abstract

**Background:**

*Astragali Radix* (AR) is widely-used for improving liver fibrosis, but, the mechanism of action has not been systematically explained. This study aims to investigate the mechanism of AR intervention in liver fibrosis based on comprehensive metabolomics combined with network pharmacology approach.

**Materials and methods:**

UPLC–Q-TOF/MS based metabolomics technique was used to explore the specific metabolites and possible pathways of AR affecting the pathological process of liver fibrosis. Network pharmacology analysis was introduced to explore the key targets of AR regarding the mechanisms on liver fibrosis.

**Results:**

AR significantly reduced the levels of ALT, AST and AKP in serum, and improved pathological characteristics. Metabolomics analysis showed that the therapeutic effect of AR was mainly related to the regulation of nine metabolites, including sphingosine, 6-keto-prostaglandin F1a, LysoPC (O-18:0), 3-dehydrosphinganine, 5,6-epoxy-8,11,14-eicosatrienoic acid, leukotriene C4, taurochenodesoxycholic acid, LysoPC (18:1 (9Z)) and 2-acetyl-1-alkyl-sn-glycero-3-phosphocholine. Pathway analysis indicated that the treatment of AR on liver fibrosis was related to arachidonic acid metabolism, ether lipid metabolism, sphingolipid metabolism, glycerophospholipid metabolism and primary bile acid biosynthesis. Validation of the key targets by network pharmacology analysis of potential metabolic markers showed that AR significantly down-regulated the expression of CYP1B1 and up-regulated the expression of CYP1A2 and PCYT1A.

**Conclusion:**

Metabolomics combined with network pharmacology was used for the first time to clarify that the treatment of AR on liver fibrosis, which is related to the regulation of arachidonic acid metabolism and ether lipid metabolism by modulating the expression of CYP1A2, CYP1B1 and PCYT1A. And the integrated approach can provide new strategies and ideas for the study of molecular mechanisms of traditional Chinese medicines in the treatment of liver fibrosis.

## Background

As a worldwide clinical problem, liver fibrosis is a wound healing process with recurrent chronic liver damage [1]. It can accelerate the development of chronic liver disease by destroying normal liver parenchyma, eventually leading to cirrhosis, liver failure and even primary liver cancer [2]. Although the pathogenesis of liver fibrosis, such as inflammatory response, hepatic stellate cell activation and extracellular matrix formation, has been widely recognized, it has not got an effective and powerful treatment [3, 4]. Therefore, it is necessary to develop an effective treatment tool for liver fibrosis with high efficiency, low side effects and multiple targets.

*Astragali Radix* (AR), is a worldwide used traditional Chinese medicine (TCM) is the dried root of *Astragali radix membranaceus* (Fisch.) Bge. or *Astragali radix membranaceus* (Fisch.) Bge. var. mongholicus (Bge) Hsiao [5, 6]. In traditional Chinese formula, AR is often used for the treatment of liver fibrosis [6, 7]. Fundamental studies have exhibited the anti-hepatic fibrosis effects of AR by inhibiting TGF-β/Smads signaling [8, 9]. In addition, AR and its active ingredients have obvious protective effects on cholestasis [8], carbon tetrachloride [10], dimethyl nitrosamine [8], acetaminophen [11] and ethanol [12] induced liver injury. Moreover, studies have shown that AR is safe without obvious toxicity, side effects or genotoxicity [13, 14]. Therefore, AR exhibits significant advantages for the treatment of liver fibrosis.

Metabolomics can characterize the dynamic changes of metabolites throughout the biological system providing a powerful platform for discovering new biomarkers and biochemical pathways [15, 16], improving diagnosis [17], treatment and prediction [18, 19] in complex systems. Although some studies have analyzed the metabolites of AR in vivo, only the changes of endogenous metabolites of AR from different habitats in normal mice for quality evaluation were compared [5, 20]. The analysis of metabolite changes of AR in diseased mice is currently lacking. In addition, network pharmacology has become a powerful tool for studying complex diseases to reveal the complex relationships between proteins, diseases and drugs [21]. This method helps to determine the main active ingredients of drugs and their role in the treatment of various diseases [22, 23]. A combination of metabolomics and network pharmacology can link endogenous metabolites to disease targets, further to uncover the molecular mechanisms of TCM with multi-component and multi-target characteristics [24].

**Fig. 1:**
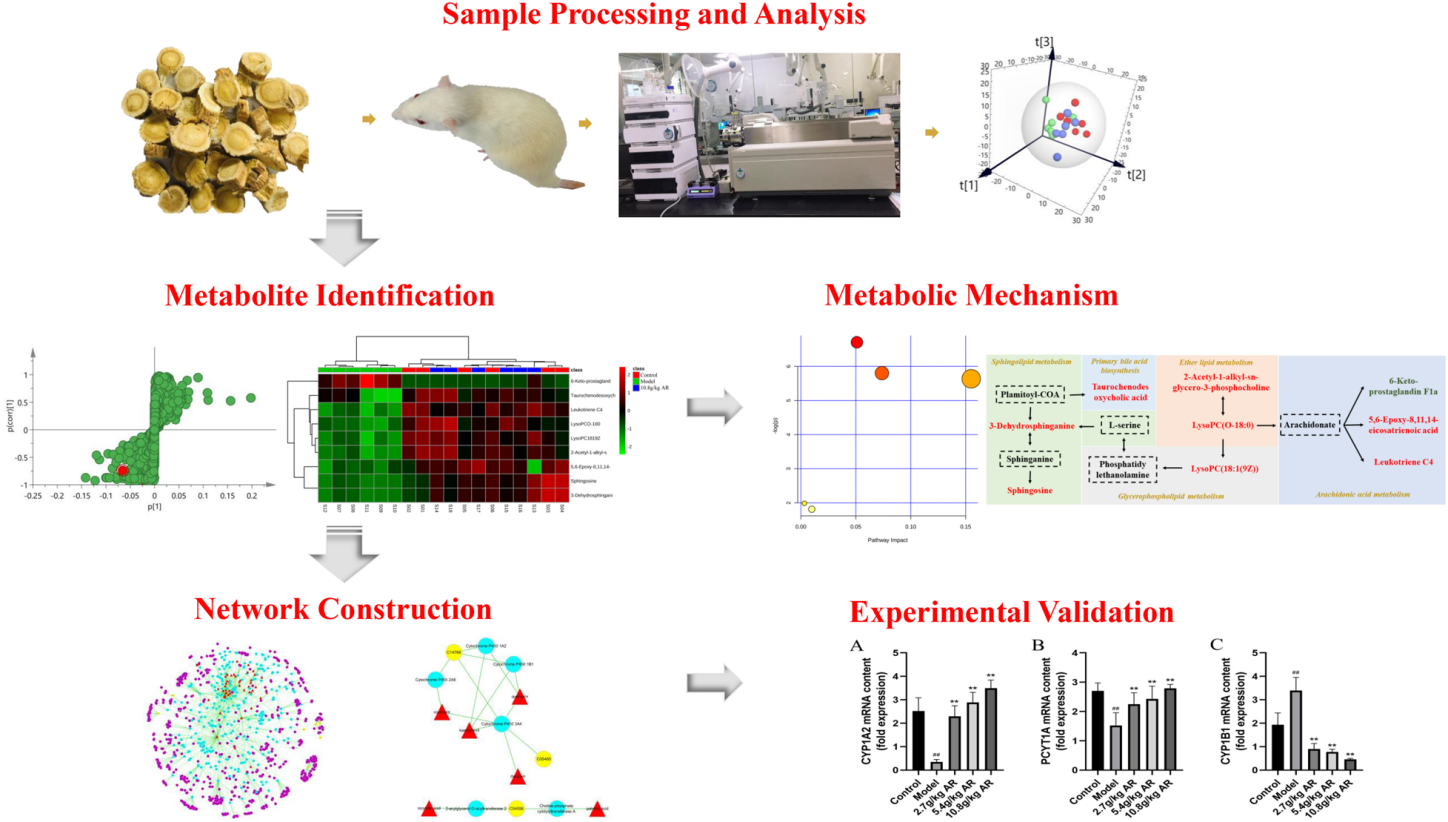
Scheme of the study

This study combined the UPLC–Q-TOF/MS serum metabolomics and network pharmacology techniques to systematically explain the modulatory properties of AR on liver fibrosis. Multivariate data analysis was used to screen potential metabolite makers and corresponding metabolic pathways to explore the function of AR. The feasibility of AR for the treatment of liver fibrosis was further confirmed by constructing a component–target–metabolite network to identify the key targets on liver fibrosis (Fig. 1).

## Methods

### Reagents

Colchicine was got from XiShuangBanNa BanNa Pharmaceutical Co. (Yunnan, China). Carbon tetrachloride (CCl_4_) was purchased from Tianjin Guangfu Chemical Research Institute (Tianjin, China). Alanine amino transferase (ALT), aspartate amino transferase (AST), alkaline phosphatase (AKP) detection kits were bought from Nanjing Jiancheng Bioengineering Institute (Nanjing, China). alpha smooth muscle actinα (α-SMA) and transforming growth factor-beta (TGF-β1) were bought from Cell Signaling Technology (United States). GAPDH was bought from ABclonal (China). Quercetin and nicotinic acid were bought from Chengdu Cloma Biological Co., Ltd (China).

### Preparation of AR water extract

AR (the dried root of *Astragali radix membranaceus* (Fisch.) Bge.) was purchased from Beijing Lvye pharmaceutical co. (Beijing, China). Firstly, AR was extracted with boiling water (1/10, *w/v*) for 2 h. Then, it was extracted twice with boiling water (1/8, *w/v*) for 1.5 h each time. The aqueous extract of AR was dried to power by freeze vacuum drying oven, and the final yield of powder was about 40.08%. A Q-TOF LC/MS analysis was carried out to identify the main constituent in the tested extract (Additional file 1: Figure S1 and Table S1).

The active ingredients of AR extract were identified on Triple TOF 4600 high-resolution mass spectrometry system (AB SCIEX, USA), and 3 μL of each sample was injected into ZORBAX Eclipse C8 analytical column (1.8 μm i.d., 2.1 mm i.d. × 100 mm, Agilent Technologies, USA). The column temperature was maintained at 35 °C. The flow rate was set as 0.25 mL/min. the mobile phase was solvent A (water with 0.1% formic acid) and solvent B (acetonitrile with 0.1% formic acid). The gradient was used as follows: a linear gradient of 0% B over initial-1.0 min, 0–5% B over 1.0–8.0 min, 5–55% B over 8.0–20.0 min, 55% B in 20.0–26.0 min and 55–5% B over 26.0–30.0 min. The eluent was directly introduced into the mass spectrometer. The mass spectrometry conditions were as follows: the electrospray capillary voltage was 5.5 kV in positive ionization mode and 4.5 kV in negative ionization mode. The Gas1 and Gas2 were 55 Psi, the gas temperature was 600 °C, and the collision voltage was 40 V in the positive and negative ionization mode. The full scan mode was adopted and with the aid of information correlation acquisition and background subtraction technology. The first scanning frequency was 0.2 s and the second scan frequency was 0.12 s.

### Animal experiments and sample collection

180–200 g male Sprague–Dawley rats were offered by the China food and drug certification research institute (Permission NO. SCXK(Jing)-2014-0013) and housed with same feeding environment (temperature: 25 °C ± 2 °C, humidity: 55% ± 5% 12:12 h light: dark cycle) in the Central Animal Laboratory of The Fifth Medical Center of PLA General Hospital.

Thirty-six rats were randomly divided into six groups. The control group was only given with olive oil and the other five groups were given with intraperitoneal administration of olive oil (1:1, *v/v*) dissolved CCl_4_ (2 mL/kg) twice a week for 8 weeks. Then AR (10.8, 5.4, 2.7 g/kg/day) and colchicine (0.2 mg/kg/day, as a positive control group) were administered by intragastric administration for 6 weeks, except for the control and model groups, and CCl_4_ was given intraperitoneally at the same time.

After 12 h of the last treatment, the rats were sacrificed and liver and blood were collected. The blood was centrifuged at 3500 rpm for 15 min to separate the serum without hemolysis. Serum and liver tissue were stored at − 80 °C for the biochemical parameters and metabolomics analysis. All animal experiments were approved by the Ethical Committee of Fifth Medical Center of PLA General Hospital of China.

Serum levels of ALT, AST and AKP were tested according to the instruction of kits. Liver tissue was fixed in 10% neutral buffered formalin for 24 h. All fixed livers were embedded in paraffin, and then were cut into sections (about 4–5 μm thick) by using a microtome. Hematoxylin–eosin (HE) and Masson staining were used for highlighting the liver damage and collagen deposition, respectively.

### Immunofluorescence and immunohistochemistry analysis

To measure immunofluorescence, liver tissues were sliced into 14 μm thick sections and then blocked with blocking buffer containing 0.01 M phosphate buffered saline (PBS), 0.1% Triton X-100 and 5% normal goat serum solution for 60 min. Subsequently, the sections were incubated with primary antibody [TGF-β1 (1:100)]. The sections were washed and incubated with an anti-rabbit Alexa Fluor 488-conjugated IgG secondary antibody. 4,6-diamidino-2-phenylindole counterstaining was used to stain the nuclei. The sections were cover-slipped with fluorescent mounting medium.

To measure immunohistochemical, liver tissues were immersed in 2% H_2_O_2_ for 25 min at room temperature and then blocked with 5% rabbit serum for 30 min. Then, the primary antibody [α-SMA (1:100)] was added for incubation at 4 °C overnight. After washing with PBS, the liver sections were incubated with horseradish peroxidase-conjugated secondary antibody for 50 min at room temperature. Liver sections were immersed in diaminobenzidine for 3 min and then stained with ethanol dehydrated hematoxylin. The stained areas of the sections were observed under an optical microscope of 200 nm.

### Western blotting

Liver tissue (80 mg) was homogenized and lysed in ice-Cold lysis buffer with PMSF and protein phosphatase inhibitor mixture, and then centrifuged at 12,000×*g* and 4 °C for a 10 min. The supernatant was western blotted with 10% SDS-PAGE and the blot was transferred to the polyvinylidene fluoride membrane. Subsequently, it was incubated with 5% skim milk powder in blocking buffer for 2 h. And polyvinylidene fluoride membrane incubated with α-SMA (1:1000) and GAPDH (1:2000) primary antibodies at 4 °C with gentle shaking overnight. Finally, after incubation with the secondary antibody for 1 h, the membrane was washed 3 times with TBST for 5 min each time and the immunoreactivity bands were detected by chemiluminescence detection kit.

### Sample preparation and UPLC–Q-TOF/MS assay

600 μL of methanol was added to 200 μL of serum and was mixed. The sample was allowed to stand at 4 °C for 20 min and then centrifuged at 12,000 rpm for 10 min. Finally, the supernatant was absorbed and filtered through 0.22 μm micropore filter. The filtrate was collected for analysis. For the quality control (QC) samples, taken 20 μL from each prepare sample extract and mix, used the rest of the samples for Q-TOF LC/MS test.

An Agilent 6550 iFunnel Q-TOF LC/MS (Agilent Technologies, USA) system was used for serum metabolic spectrum analysis. A 4 μL aliquot of each sample was injected into the system on a ZORBOX RRHD C18 analytical column (2.1 mm i.d. × 100 mm, 1.8 μmi.d., Agilent Technologies, USA) for sample separation at 30 °C. Solvent A (water containing 0.1% formic acid) and solvent B (acetonitrile containing 0.1% formic acid) were used as the mobile phase for a linear gradient separation at a flow rate of 0.30 mL/min for 25 min a linear gradient of 100% A over 0–1.0 min, 100–60% A over 1.0–9.0 min, 60–10% A over 9.0–19.0 min, 10–0% A over 19.0–21.0 min, 100% B over 21.0–25.0 min.

Both positive and negative mode electrospray ionization sources (ESI) were used. The electrospray source parameters are set as follows: electrospray capillary voltage is 3.5 kV in negative ionization mode and 4 kV in positive ionization mode, mass range is from *m/z* 50 to 1200, gas temperature is 225 °C, gas flow rate is 13 L/min, nebulizer is set to 20 psi, sheath gas temperature is 275 °C, sheath gas flow is 12 L/min, and nozzle voltage is 2000 V in both negative and positive modes.

### Data extraction and multivariate analysis

MassHunter Profinder software (Agilent, California, United States) was used to extract sample data and perform peak detection and alignment. The full scan mode is applied to the mass range of *m/z* 80–1000 and sets the initial and final retention times for data collection. Data was normalized using MetaboAnalyst 4.0 and then analyzed by principal component analysis (PCA) and orthogonal-partial least squares-discriminant analysis (OPLS-DA) using SIMCA-P 14.1 software (Umetrics, Umea, Sweden).

### Biomarkers identification and pathway enrichment analysis

Biomarkers were discovered by screening for metabolic differences. Differential metabolite satisfying the conditions (VIP>1.0, |p(corr)| ≥ 0.58 and *P*<0.05 in ANOVA) were used as potential biomarkers in the OPLS-DA analysis [24]. Metabolites (molecular weight error < 20 ppm) were identified based on precise molecular weight in the Human Metabolome Database (HMDB) and Metlin database. The identified compounds were resubmitted to MetaboAnalyst 4.0 to analyze their signaling pathways.

### Identification of drug targets and network construction

The chemical components of AR (oral bioavailability (OB) ≥ 30% and a drug-likeness (DL) > 0.18) were collected in the TCMSP database. And the MBROLE 2.0 database was used to collect the protein targets for potential metabolites. UniProt ID was used to replace different types of protein IDs, and then a metabolic–target–component interaction network was established through protein interaction (PPI) information. Finally, the network was visualized and analyzed using Cytoscape 3.6.1 software.

### Real-time polymerase chain reaction (RT-PCR) detection

Total RNA was extracted from liver tissue by trizol reagent and RNA (2 μg) was transcribed into cDNA by PrimerScript RT regent kit. The cDNA was subsequently subjected to PCR amplification by ABI Step One Plus. Data analysis was performed by 2^−ΔΔCT^ method. The primers are listed in Table 1.

**Table 1:** Primer sequences used for RT-PCR

Gene	Forward	Reverse
CYP1A2	GTGGTGGAATCGGTGGCTAATGTC	CTTGCTGCTCTTCACGAGGTTGAG
CYP1B1	CGAGAGTTGGTGGCAGTGTTGG	CTCGGCATCGTCGTGGTTGTAC
PCYT1A	AGACGAGGTGGTGAGGAACGC	TGGAGATGCCTTCTGTCCTCTGTG
α-SMA	GGCCACTGCTGCTTCCTCTTC	TGCCCGCCGACTCCATTCC
TGF-β1	ATGGTGGACCGCAACAACGC	CTGGCACTGCTTCCCGAATGTC
GAPDH	GTCCATGCCATCACTGCCACTC	GATGACCTTGCCCACAGCCTTG

### Statistical analysis

Data were analyzed by one-way ANOVA and Duncan’s multi-range test. The SPSS computer program was used to analyze the mean of Windows. Results are expressed as mean ± standard deviation (SD). *P* < 0.05 was considered statistically significant.

## Results

### AR reduced pathological damage in CCl_4_-induced liver fibrosis

**Fig. 2:**
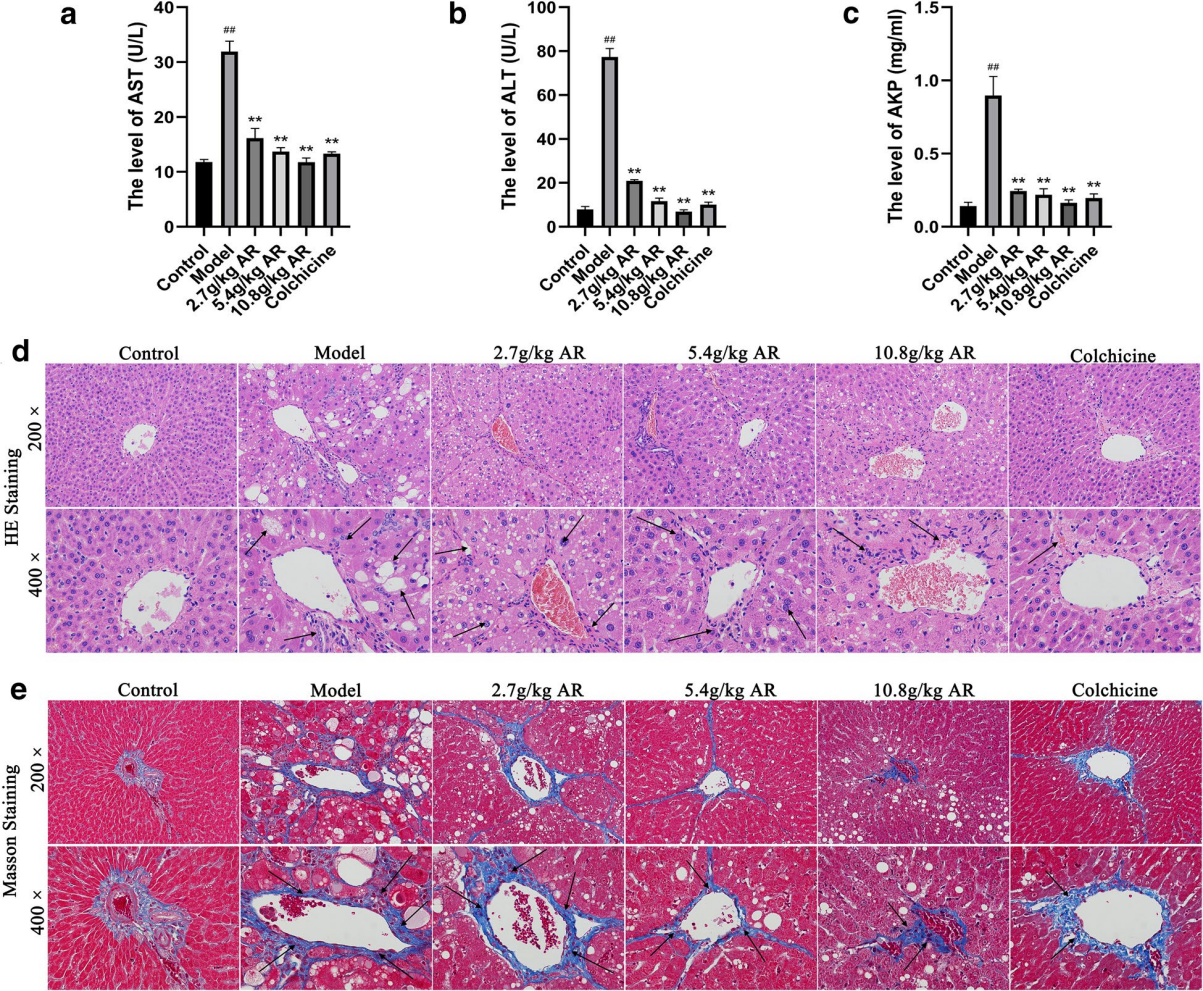
Effect of AR on liver function and histopathology of liver fibrosis. **a** AR decreased serum AST level. **b** AR decreased serum ALT level. **c** AR decreased serum AKP level. **d** Hematoxylin and eosin (HE) stained liver section in six groups. Original magnification, ×200, ×400. **e** Histological examination of liver section with Masson stain. Original magnification, ×200, ×400. Blue areas show collagen fibers and damaged liver tissue. Data were expressed as mean ± SD (n = 6). ^#^*P *< 0.05, ^##^*P *< 0.01 compared with control group; *P < 0.05, **P < 0.01 compared with model group. *ALT* alanine amino transferase, *AST* aspartate amino transferase, *AKP* alkaline phosphatase

As shown in Fig. 2a–c, serum ALT, AST and AKP levels in the model group were significantly increased (*P* < 0.01) compared with that in the control group, respectively. And serum ALT, AST and AKP concentrations were attenuated in a dose-dependent manner by AR. Compared with the model group, ALT, AKP and AST levels were most significantly reduced after administration of 10.8 g/kg AR alone (*P* < 0.01). Histologic analysis was performed using HE staining and MASSON staining. As shown in Fig. 2d, the liver structure of rats in the control group was normal, and several histological features of the liver in CCl_4_-administration group included pericentral necrosis and fibrosis, vacuolar steatosis, inflammatory cell infiltration, and cytoplasmic degeneration. AR treatment of animals exposed to CCl_4_ can effectively improve liver necrosis, fibrosis, and reduce inflammatory infiltration, especially in 10.8 g/kg AR group. In the liver section of MASSON staining, the blue area represented the deposited collagen. The results showed that a large amount of collagen was deposited around the hepatic sinus of the model group. And compared with model group, 10.8 g/kg AR could effectively reduce collagen deposition and improve liver fibrosis (Fig. 2e).

### AR reduced CCl_4_-induced liver fibrosis

**Fig. 3:**
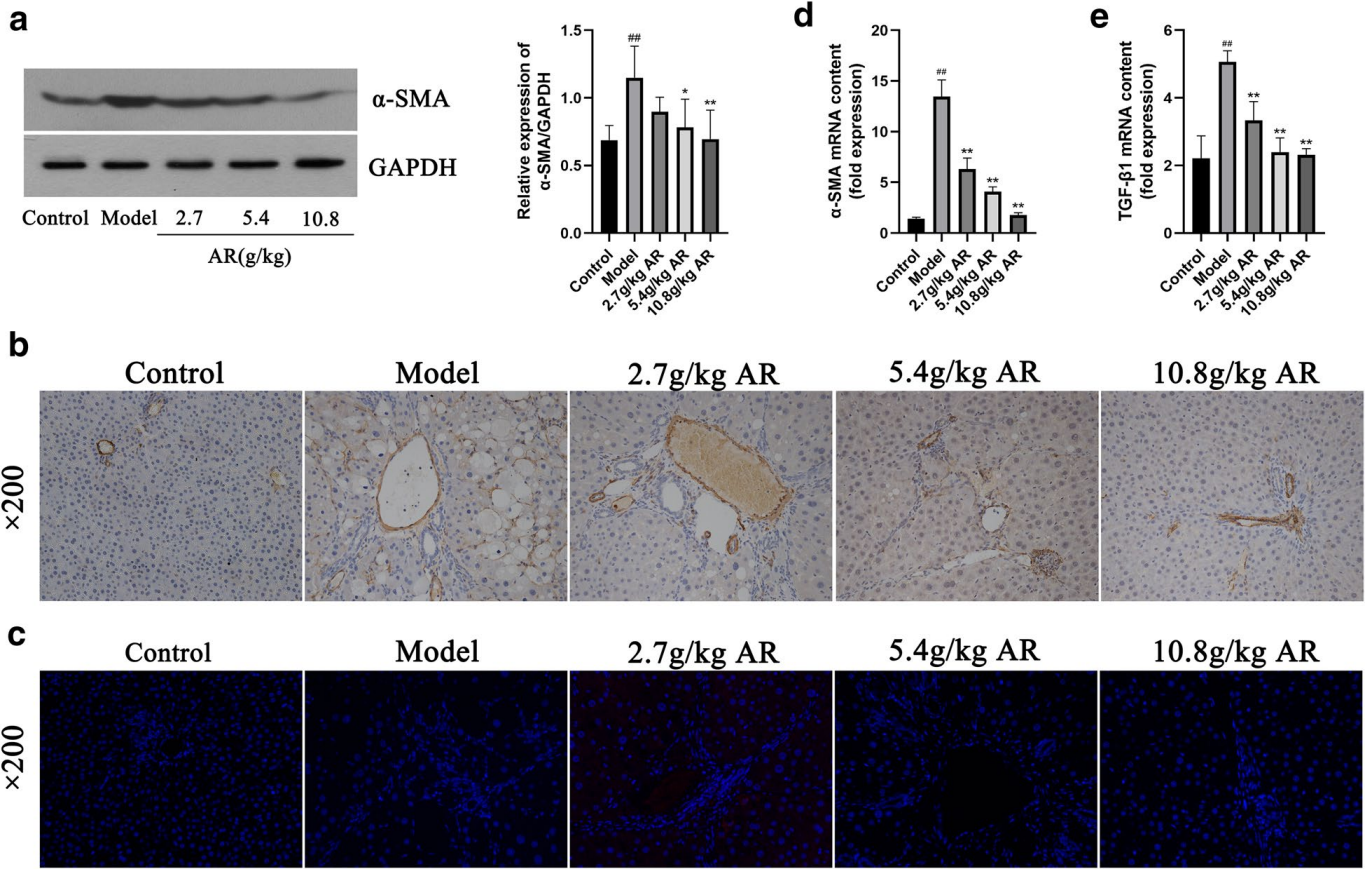
AR alleviated α-SMA and TGF-β1 expression during liver fibrosis. **a** Western bolt analysis for α-SMA. **b** Immunohistochemical analysis for α-SMA. **c** Immunofluorescence analysis for TGF-β1. **d** RT-PCR analysis for α-SMA. **e** RT-PCR analysis for TGF-β1. Data were expressed as mean ± SD (n = 6). ^#^*P *< 0.05, ^##^*P *< 0.01 compared with control group; *P < 0.05, **P < 0.01 compared with model group. *α-SMA* alpha smooth muscle actin, *TGF-β1* transforming growth factor-beta

α-SMA and TGF-β1 are two key markers of CCl_4_-induced liver fibrosis. Western blot, immunohistochemistry and immunofluorescence were used to analyze the correlation between AR and liver fibrosis. As shown in Fig. 3a, western blotting showed that the expression of α-SMA protein in the model group was significantly higher than that in the control group. And α-SMA expression was decreased in a dose-dependent manner by AR. These results were consistent with the results observed via immunohistochemistry (Fig. 3b). Immunofluorescence showed that TGF-β1 expression in model group increased significantly and the expression of TGF-β1 was lower than that in the model group after administration of AR, especially in the 10.8 g/kg AR group (Fig. 3c). Furthermore, the mRNA levels of α-SMA and TGF-β1 were detected by RT-PCR. The results of RT-PCR confirmed the above results again (Fig. 3d, e). In conclusion, these results suggest that AR can reduce CCl_4_-induced liver fibrosis in a dose-dependent manner. Therefore, 10.8 g/kg of AR was selected as the optimal effective dose for metabolic analysis and subsequently screening for metabolic differences.

### Quality control

**Fig. 4:**
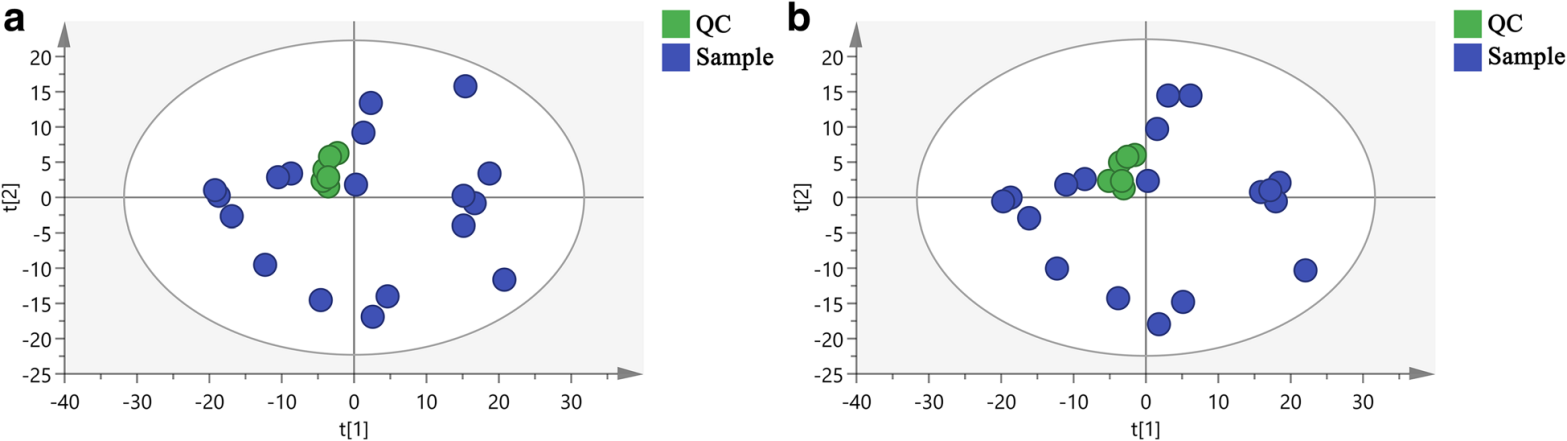
Principal component analysis (PCA) score plot of quality control (QC) samples. **a** ESI+ mode. **b** ESI− mode

QC can determine whether the systematic error of the whole experiment is within the controllable range. As shown in Fig. 4, the cluster analysis of QC samples relative to the experimental samples showed that the QC samples were closely clustered, especially in positive ion mode. The above results prove that the method has good stability and repeatability.

### Multivariate statistical analysis

**Fig. 5:**
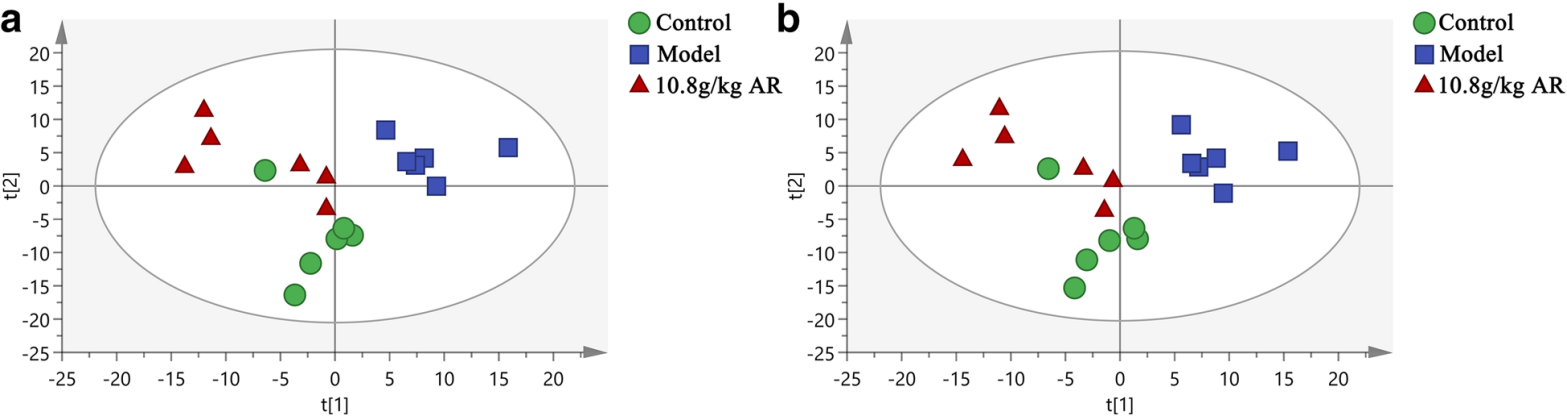
Principal component analysis (PCA) score plot of normal, model and 10.8 g/kg AR groups. **a** ESI+ mode. **b** ESI− mode

Firstly, SIMCA-14.1 software was used to distinguish the control, model and 10.8 g/kg of AR groups in the serum metabolic phenotypes and metabolites by PCA. The purpose of PCA was description, which is used to observe the separation between different groups of metabolites [25, 26]. The results showed that a significant classification between the clustering of the control and model groups and the control and AR groups was observed in both the ESI+ and ESI− modes (Fig. 5). As shown in the Additional file 2: Figure S2, the corresponding loading scores plots from PCA could well summarize the influence of variables on the model. Subsequently, multivariate analysis was used to explore which metabolites contributed to these differences.

**Fig. 6:**
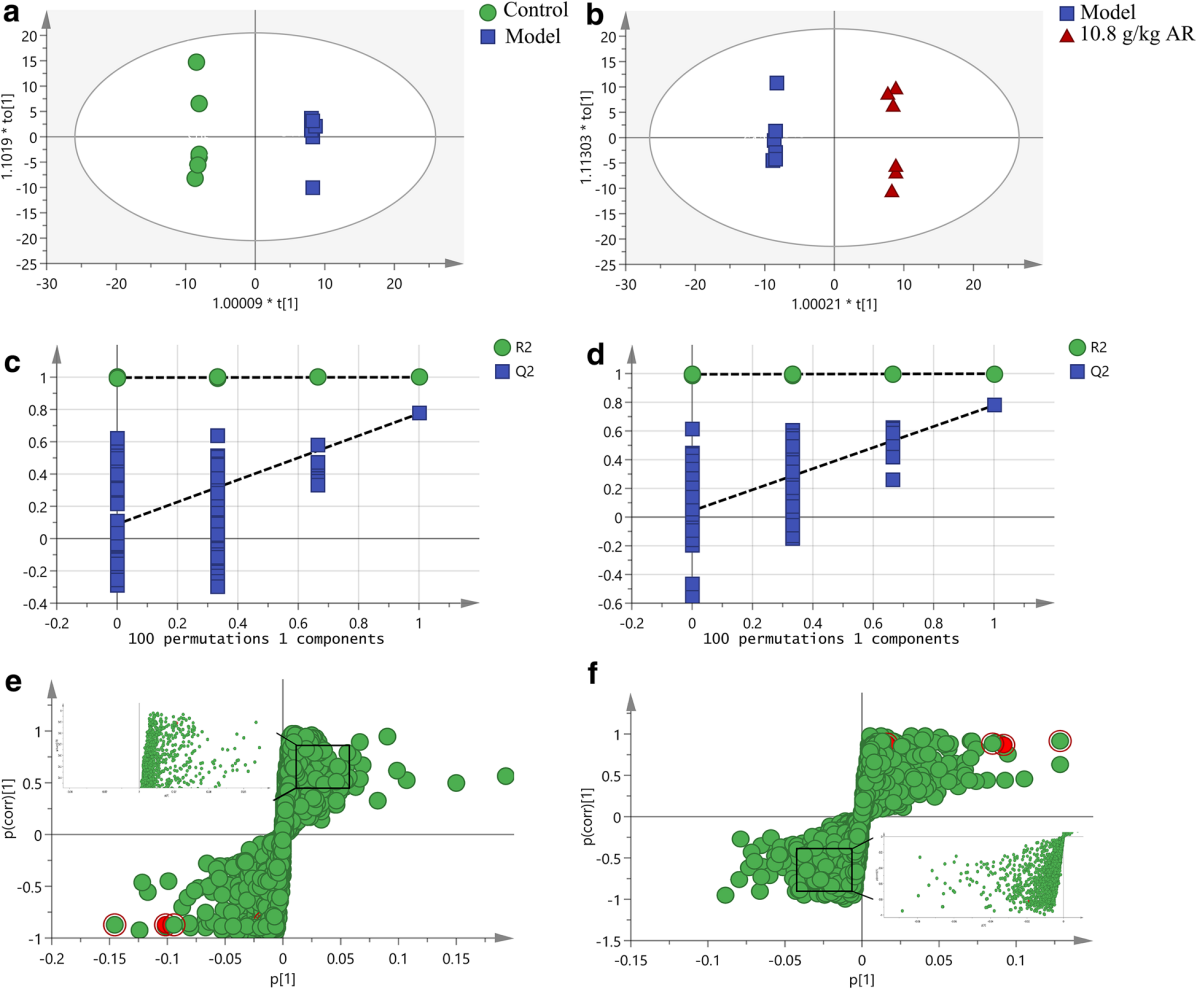
The OPLS-DA score plots, S-plots and 100-permutation test generated in ESI+ mode. OPLS-DA score plots were the pair-wise comparisons between the control and model groups (**a**) as well as between the model and AR groups (**b**). The 100-permutation test of the OPLS-DA mode was for the control and model groups (**c**) as well as for the model and AR groups (**d**). S-plots of the OPLS-DA mode for the control and model groups (**e**) as well as for the model and AR groups (**f**)

OPLS-DA, as a pattern recognition approach, was designed for predictions to identify differential metabolites that were significantly changed between control, model and 10.8 g/kg of AR groups [25, 26]. The OPLS-DA could only be used for screening differentially expressed metabolites between the two groups [27]. And this study aimed to explore the specific metabolites regulated by AR in rats with liver fibrosis. Therefore, we respectively identified differential metabolites from control vs model groups and model vs 10.8 g/kg AR groups in OPLS-DA mode. Metabolites identified between the different groups are listed in Additional file 3: Tables S2 and S3. The same differential metabolites were chosen for subsequent analysis. In the OPLS-DA mode established from the serum data of the control and model groups, the variance of the response variable (R^2^Y) in the positive and negative modes were 1 and 1, and the variance for modeling in cross-validations (Q^2^) were 0.774 and 0.777, respectively (Fig. 6a and Additional file 4: Figure S3A). For serum data of the model and AR groups in positive and negative modes, R^2^Y values were 0.999 and 0.999 with Q^2^ 0.778 and 0.778, individually (Fig. 6b and Additional file 4: S3B). The relevant parameter demonstrated that the modes had good explanatory and predictive ability. The permutation tests (*n *= 100) were performed to validate the predictive ability of modes (Fig. 6c, d and Additional file 4: S3C, D). The results showed that all R^2^ and Q^2^ values were lower than that in the permutation tests, demonstrating the goodness of fit and better predictability of the OPLS-DA mode. The S-plot was used to study the inherent clustering variables. In the corresponding S-plot, variables whose VIP value were over 1 at average and P(corr) absolute values over 0.58 means can be considered as potential biomarkers (Fig. 6e, f and Additional file 4: S3E, F). In order to characterize differential metabolites more comprehensively, the potential differential metabolites obtained under ESI+ and ESI− modes were combined for subsequent analysis [28].

### Identification of potential metabolites in AR treatment

**Fig. 7:**
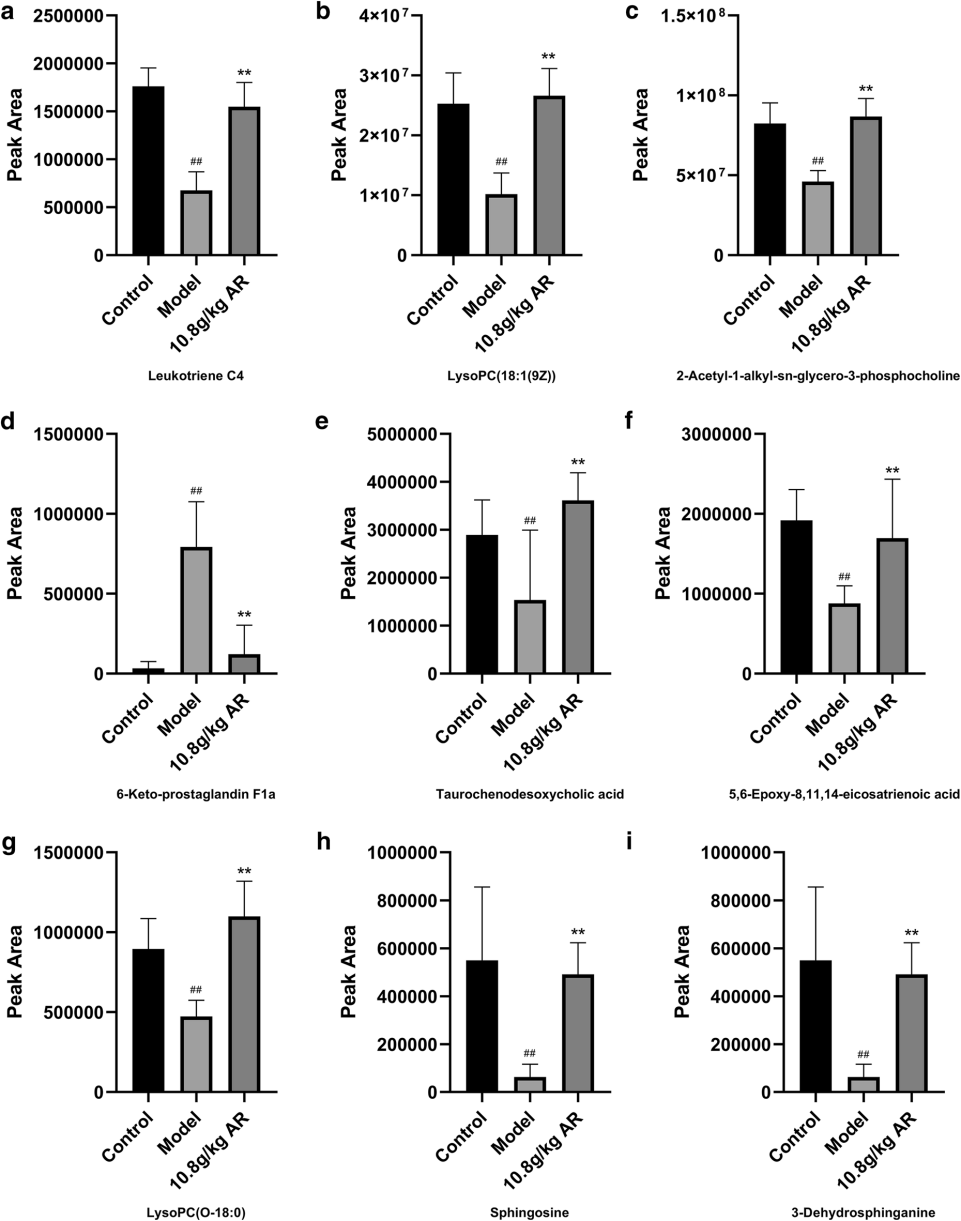
Potential metabolites changes in CCl_4_-induced liver injury treated by AR. **a** Leukotriene C4; **b** LysoPC(18:1 (9Z)); **c** 2-Acetyl-1-alkyl-sn-glycero-3-phosphocholine; **d** 6-Keto-prostaglandin F1a; **e** Taurochenodesoxycholic acid; **f** 5,6-Epoxy-8,11,14-eicosatrienoic acid; **g** LysoPC(O-18:0); **h** Sphingosine; **i** 3-Dehydrosphinganine. Data were expressed as mean ± SD (n = 6). ^#^*P *< 0.05, ^##^*P *< 0.01 compared with control group; *P < 0.05, **P < 0.01 compared with model group

**Fig. 8:**
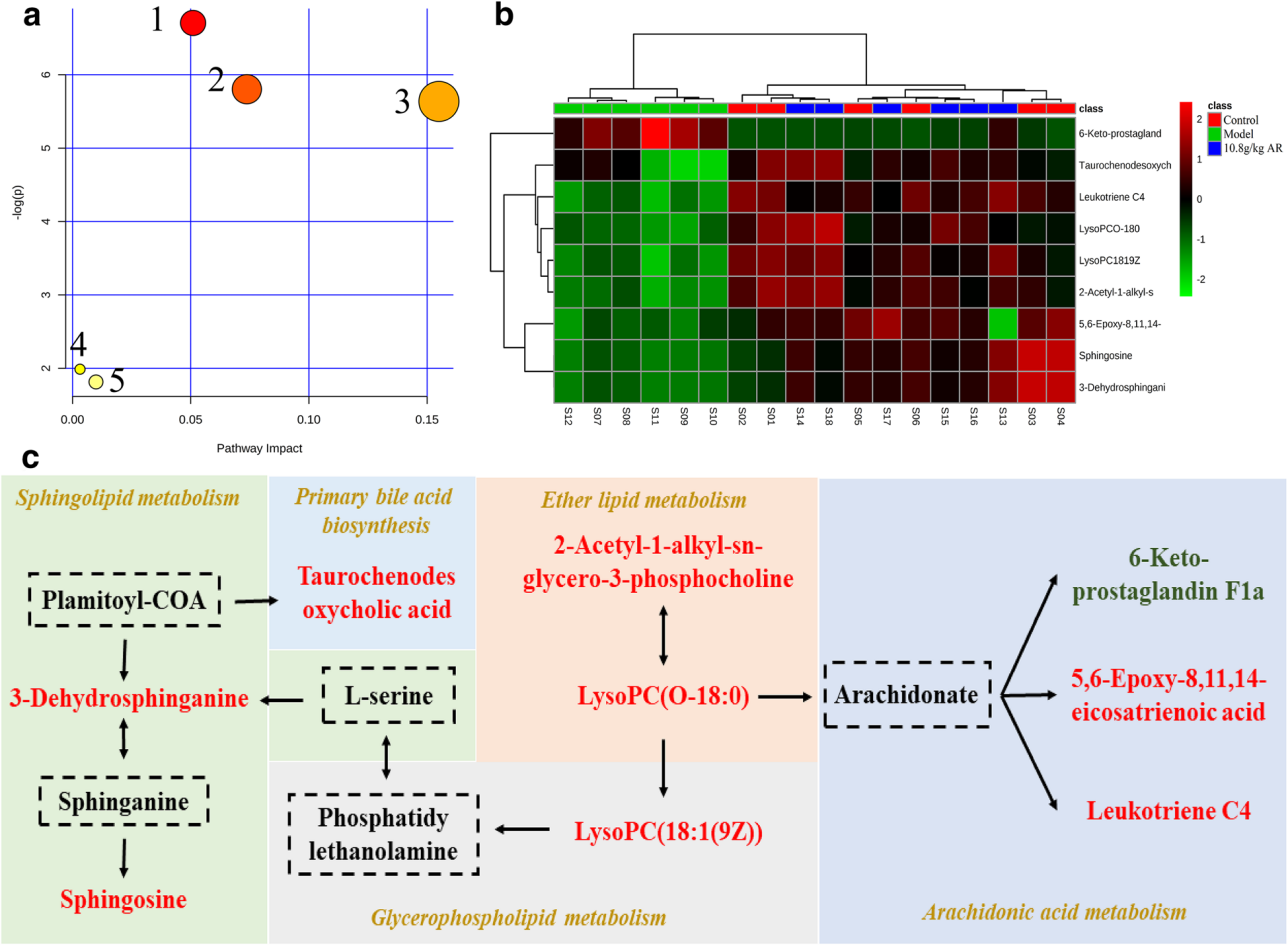
Potential metabolomic pathway in CCl_4_-induced liver injury treated by AR. **a** Metabolomic pathway construction of the metabolic pathways involved in the effects of AR on liver fibrosis. **b** The heatmap of 9 potential metabolites. **c** Signaling networks associated with the differentially expressed metabolic pathways. 1: Arachidonic acid metabolism. 2: Ether lipid metabolism. 3: Sphingolipid metabolism. 4: Glycerophospholipid metabolism. 5: Primary bile acid biosynthesis

During the potential metabolite identification, 595 potential differential metabolites were selected based on the principles that VIP > 1.0 and |P(corr)| ≥ 0.58 in the S-plots as candidates for ANOVA analysis. Candidate variables with significant differences were then screened using ANOVA analysis. Candidates with significant changes were identified as biomarkers based on the METLIN and Metaboanalyst databases. A total of 9 metabolic differences were identified, 7 in ESI+ mode and 2 in ESI− mode, which were marked with red in the S-plots diagram (Fig. 6E, F and Additional file 4: S3E, F). As shown in Fig. 7, the nine potential biomarkers were sphingosine (C00319), 6-keto-prostaglandin F1a (C05961), LysoPC (O-18:0) (C04317), 3-dehydrosphinganine (C02934), 5,6-epoxy-8,11,14-eicosatrienoic acid (C14768), leukotriene C4 (C02166), taurochenodesoxycholic acid (C05465), LysoPC (18:1 (9Z)) (C04230), 2-acetyl-1-alkyl-sn-glycero-3-phosphocholine (C04598). The distribution patterns of 9 potential metabolites in the three groups were visually displayed by heatmap. And vertical cluster analysis was used to demonstrate the difference between model and control groups and the equivalent efficacy of AR (Fig. 8b). The corresponding formula and parameters including retention time, *m/z* and differences between groups were listed in Table 2.

**Table 2:** Identified metabolites of the serum from different groups

No.	R.T. (min)	Mass (m/z)	Metabolites	Formula	KEGG	Change trend		Pathway
						Control/model	Model/AR	
1	14.174	624.281	Leukotriene C4	C_30_H_47_N_3_O_9_S	C02166	Down	Up	Arachidonic acid metabolism
2	14.224	544.339	LysoPC(18:1 (9Z))	C_26_H_52_NO_7_P	C04230	Down	Up	Glycerophospholipid metabolism
3	16.987	524.371	2-Acetyl-1-alkyl-sn-glycero-3-phosphocholine	C_26_H_54_NO_7_P	C04598	Down	Up	Ether lipid metabolism
4	10.064	371.235	6-Keto-prostaglandin F1a	C_20_H_34_O_6_	C05961	Up	Down	Arachidonic acid metabolism
5	14.609	498.335	Taurochenodesoxycholic acid	C_26_H_45_NO_6_S	C05465	Down	Up	Primary bile acid biosynthesis
6	16.363	343.224	5,6-Epoxy-8,11,14-eicosatrienoic acid	C_20_H_32_O_3_	C14768	Down	Up	Arachidonic acid metabolism
7	16.030	532.340	LysoPC(O-18:0)	C_26_H_56_NO_6_P	C04317	Down	Up	Ether lipid metabolism
8	18.113	322.269	Sphingosine	C_18_H_37_NO_2_	C00319	Down	Up	Sphingolipid metabolism
9	18.150	322.270	3-Dehydrosphinganine	C_18_H_37_NO_2_	C02934	Down	Up	Sphingolipid metabolism

### Pathway analysis

To explore the mechanism of AR on liver fibrosis, the metabolic pathways were constructed by importing the identified potential metabolites into MetaboAnalyst 4.0. It could be concluded that five pathways including arachidonic acid metabolism, ether lipid metabolism, sphingolipid metabolism, glycerophospholipid metabolism and primary bile acid biosynthesis were responsible for regulating CCl_4_-induced liver fibrosis (Table 3). As shown in Fig. 8a, the top three pathways were arachidonic acid metabolism, ether lipid metabolism and sphingolipid metabolism, which played a key role in reflecting changes. The impact-values of metabolites were 0.051, 0.07375 and 0.15499, respectively. The MetScape and KEGG pathway analysis showed that the nine metabolic differences and five pathways were directly or indirectly related, as shown in Additional file 5: Figure S4. Based on the above results, we mapped the signaling networks associated with differentially expressed metabolic pathways (Fig. 8c).

**Table 3:** Results of integrating enrichment analysis of biomarkers with MetaboAnalyst 4.0

No.	Pathway name	Match status	*P*	− log(p)	Impact
1	Arachidonic acid metabolism	3/62	0.0012245	6.7052	0.051
2	Ether lipid metabolism	2/23	0.0030196	5.8026	0.07375
3	Sphingolipid metabolism	2/25	0.0035667	5.6361	0.15499
4	Glycerophospholipid metabolism	1/39	0.13694	1.9882	0.00317
5	Primary bile acid biosynthesis	1/47	0.16287	1.8148	0.00992

### “Potential metabolite–target–component” interactive network and analysis

**Fig. 9:**
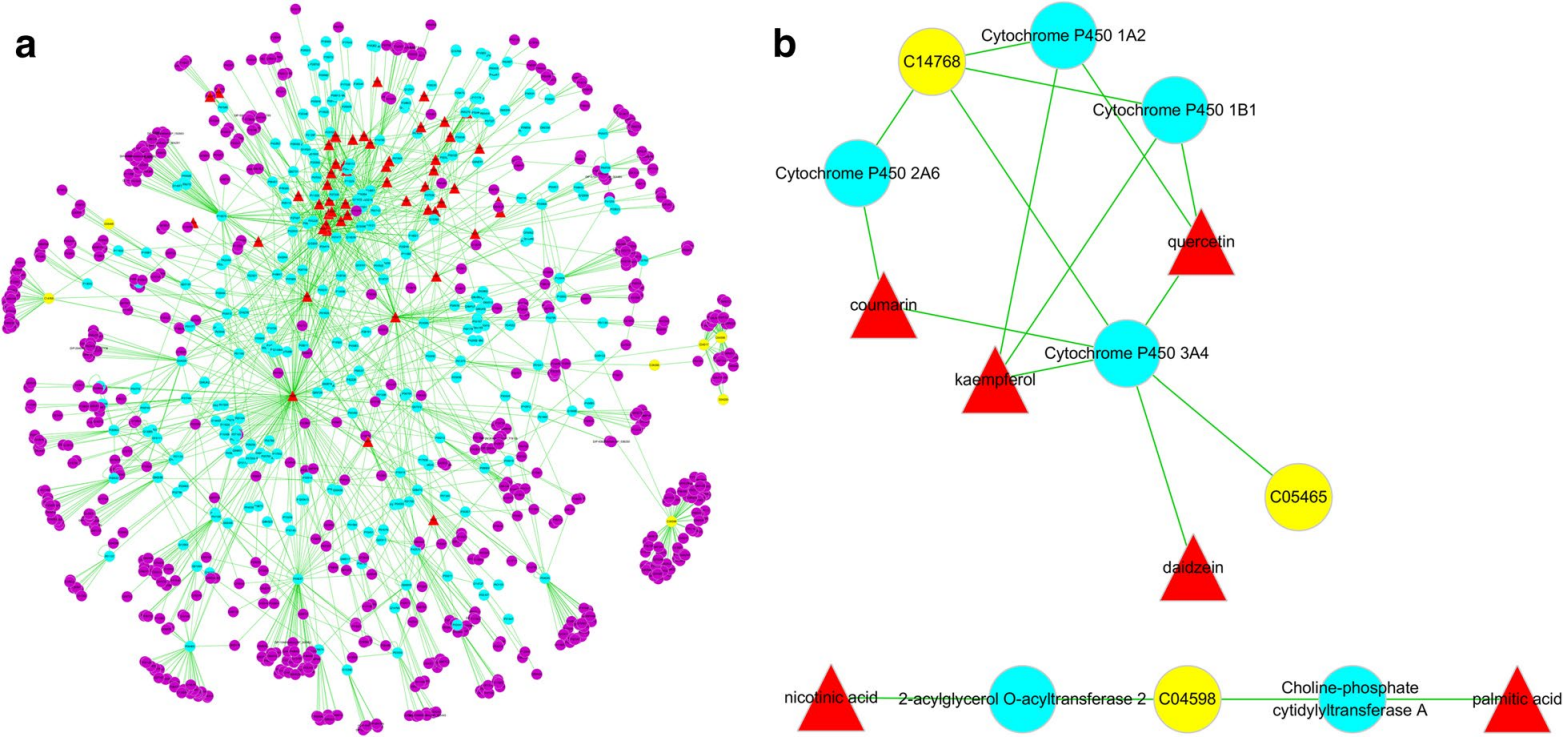
The “potential metabolite–target–component” interaction network with (**a**) all target information and (**b**) key target information participating in the treatment of liver fibrosis by AR. The red triangles represent active chemical constituents of AR. The blue dots represent the protein targets of drugs. The yellow dots represent potential metabolites. The purple dots represent the targets associated with potential metabolites

To visually reveal the interaction among the potential metabolites, protein targets and components of AR regulation, a potential metabolite–target–component interaction network was constructed by collecting drug targets and targets associated with potential metabolites. As shown in Fig. 9a, 54 components interacted with 927 targets and 8 metabolites were involved in the potential metabolite–target–component interaction network. The next analysis showed 6 drug targets, including 2-acylglycerol *O*-acyltransferase 2 (Q3SYC2), cytochrome P450 1A2 (P05177), choline-phosphate cytidylyltransferase A (P49585), cytochrome P450 3A4 (P08684), cytochrome P450 1B1 (Q16678), and cytochrome P450 2A6 (P11509), directly regulate the 3 potential metabolites (Fig. 9b). The 6 drug targets were directly regulated by the 6 chemical components of AR, including daidzein, nicotinic acid, kaempferol, coumarin, palmitic acid and quercetin (Fig. 9b).

### Verification of network pharmacology

**Fig. 10:**
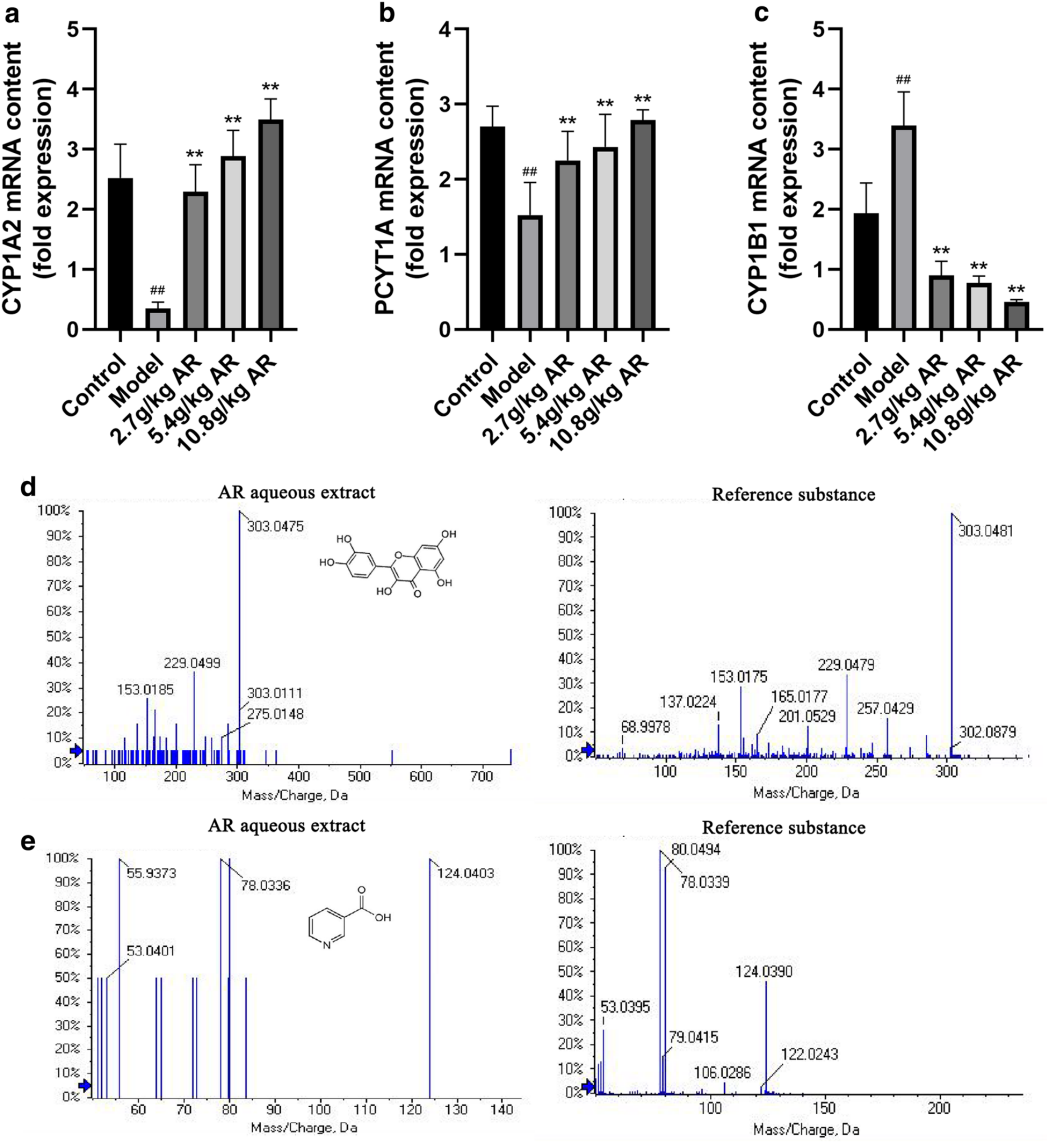
The verification results of network pharmacology. **a** The expression of CYP1A2. **b** The expression of PCYT1A. **c** The expression of CYP1B1. **d** The mass spectrum of quercetin. **e** The mass spectrum of nicotinic acid. Data were expressed as mean ± SD (n = 6). ^#^*P *< 0.05, ^##^*P *< 0.01 compared with control group; *P < 0.05, **P < 0.01 compared with model group

RT-PCR and LC–MS/MS methods were used to verify the accuracy of network pharmacology prediction results. As shown in Fig. 10a, b, the expression of CYP1A2 and PCYT1A were significantly reduced in the model group compared to the control group (*P *< 0.01). And the levels of CYP1A2 and PCYT1A were significantly up-regulated after AR treatment in a dose-dependent manner (*P *< 0.01). Furthermore, the expression of the CYP1B1 in CCl_4_-induced hepatic fibrosis rats was significantly inhibited after AR treatment (*P *< 0.01, Fig. 10c). In Fig. 10d, e, the mass spectrometry data of AR aqueous extract and reference substance showed that quercetin and nicotinic acid existed in AR aqueous extract, and the related parameters were shown in Table 4.

**Table 4:** The parameters of the active compounds in the AR extract

No.	Compound	Formula	Extraction mass	Found mass	Error (ppm)	RT (min)
1	Quercetin	C_15_H_10_O_7_	303.04993	303.04926	− 2.2	9.32
2	Nicotinic acid	C_6_H_5_NO_2_	124.03930	124.03934	0.3	2.9

## Discussion

Liver fibrosis is a chronic progressive liver disease with complex pathological mechanisms and there are no safe and effective treatment tools [1, 4]. The mean of combining metabolomics with network pharmacology to dig data extensively can effectively overcome these problems [24, 29]. Metabolomics analyzes and detects the levels of small molecules in body fluids to determine which compounds have significant abnormalities and to reveal the mechanisms of disease development [30]. Network pharmacology reveals the molecular mechanism of drug therapy by analyzing the interactions between chemical components and disease-related macromolecule targets [23]. Therefore, in this study, UPLC–Q-TOF/MS-method based rat serum metabolomics combined with network pharmacology was firstly used to clarify the protective effect of AR on liver fibrosis, which also provided an in-depth understanding of the mechanism of AR on liver fibrosis.

Serum biochemical indicators showed that AR significantly reduced serum ALT, AST and AKP levels, restored liver function and effectively alleviated liver injury. HE staining and Masson staining also confirmed that AR could effectively reduce liver damage and collagen deposition in liver tissue, as well as improved liver fibrosis. In addition, this study used a variety of methods to detect the expression of two specific makers of liver fibrosis, including α-SMA (a specific marker of hepatic stellate cell activation) and TGF-β1 (an important factor of hepatic fibrosis induced by CCl_4_) [31]. The results confirmed that AR could effectively reduce the expression of the two markers. The above results confirmed that AR exhibited significant anti-hepatic fibrosis effect, which was consistent with clinical observation.

Subsequently, the metabolomic profiles of AR in the treatment of liver fibrosis were described. AR protected liver fibrosis by reversing potential metabolites to normal levels. Biomarkers can be an effective method for disease diagnosis, treatment and prognosis [32]. Metabolism biomarkers were identified in the OPLS-DA mode. There were significant differences of biomarkers between the control group and the model group, which indicated that the serum metabolomics profile was significantly affected by CCl_4_. At the same time, significant differences were observed between the AR group and the model group, which indicated the regulatory role of AR. Finally, nine differential metabolites were screened to reveal the regulatory mechanism of AR in ameliorating liver fibrosis. These metabolites can interact in different ways. The results suggest that the development and progression of liver fibrosis were caused by the changes in many physiological and pathologically related molecules, and most of the changes in the body are interrelated. AR modulated these nine biomarkers to normalize their expression levels, indicating that AR can treat liver fibrosis through multiple pathways and multiple targets.

Those metabolites involved five main metabolic pathways including arachidonic acid metabolism, ether lipid metabolism, sphingolipid metabolism, glycerophospholipid metabolism and primary bile acid biosynthesis. The decomposition and metabolism of arachidonic acid plays a major role in triggering and eliminating inflammation [33]. Sustained inflammatory response is the key to the progress of liver fibrosis. Studies have shown that AR can improve liver fibrosis by regulating arachidonic acid metabolism. Ether lipids are a unique class of glycerophospholipids, which account for about 20% of mammalian total phospholipids and are present in small amounts in the liver [34]. Studies have shown that ether lipid metabolism was associated with liver damage caused by steatosis. Acetaldehyde phosphatide may prevent steatosis and non-alcoholic steatohepatitis through anti-oxidative stress [35, 36]. Sphingolipid metabolites played a key role in inflammatory signal. Studies have confirmed that sphingolipid metabolism was associated with hepatic lipid degeneration and inflammation [37]. Metabolic results suggested that AR played a protective role in liver by regulating sphingolipid metabolism. In addition, studies have proved that both glycerophospholipid metabolism and primary bile acid biosynthesis were closely related to liver injury. Glycerophospholipid metabolism played a key role in liver injury [38]. Bile acid is synthesized by cholesterol in the liver, and its synthesis and secretion can reflect liver function. In addition, bile acid can destroy cell membranes through its decontamination and promote oxidative stress through reactive oxygen species production, leading to hepatocyte and non-parenchymal cell damage [39].

In order to better understand the mechanism of AR in the treatment of liver fibrosis and the correlation between the AR chemical constituents and metabolites, a metabolite–target–component interaction network in combination with network pharmacology was further constructed. The results showed that six chemical components directly acted on a variety of targets, including CYP1A2, PCYT1A, CYP3A4, CYP2A6, MOGAT2 and CYP1B1, and were directly related to a variety of metabolites. CYP1A2 is a member of the CYP450s family and plays a crucial role in liver metabolism [40]. It has been found that the activity of CYP1A2 decreases with the degree of fibrosis in non-tumorous liver tissues [41]. CYP1B1 is also a member of CYP450s, which exists in hepatic endothelial cells and activated stellate cells and is involved in the metabolism of many important physiological compounds [42]. Studies have shown that hepatic steatosis and tumorigenesis could be reduced by inhibiting CYP1B1 [43]. Metabolomics combined with network pharmacological analysis showed that CYP1A2 and CYP1B1 directly regulate 5,6-Epoxy-8,11,14-eicosatrienoic acid, which participates in arachidonic acid metabolism. These results demonstrated that AR might ameliorate liver fibrosis by regulating the liver CYP450s in vivo for the potential therapeutic control of arachidonic acid metabolism. PCYT1A is the rate-limiting enzyme in the synthesis of phosphatidylcholine (PC), and PC is an essential component in all cell membranes. Previous studies have reported that pcyt1a^−/−^ mice exhibited severe fatty liver, dyslipidemia and other phenotypic characteristics [44]. MOGAT2 plays an important role in the process of fat absorption and metabolism. It is reported that *mogat2*^−/−^ mice are protected from hepatic steatosis induced by high-fat diet [45, 46]. Network pharmacological analysis showed that PCYT1A and MOGAT2 act directly on 2-Acetyl-1-alkyl-sn-glycero-3-phosphocholine, which participates in ether lipid metabolism. And these results indicated that AR could modulate ether lipid metabolism to ameliorate CCl_4_-induced hepatic steatosis and fibrosis by regulating PCYT1A and MOGAT2.

The results of RT-PCR in liver tissue of rats with liver fibrosis showed that the expression of CYP1A2 and PCYT1A was up-regulated and the expression of CYP1B1 was down-regulated in AR treatment groups, which was consistent with the previously reported results. The mRNA expression of CYP3A4, CYP2A6 and MOGAT2 was not detected, probably because the results of network pharmacology came from big data, and not all results could be verified. It might also be due to improper detection methods. Mass spectrometry data indicated that AR extract contained quercetin and nicotinic acid, which might play an important role in the treatment of liver fibrosis. The other four components were not detected, which might be due to the change of compounds during AR extraction. It might also be that the content of those components in AR extract was too low to be detected. These results not only indicated that AR could ameliorate liver fibrosis by regulating the expression of CYP1A2, PCYT1A and CYP1B1, but also confirmed that the predicted results of network pharmacology were credible.

Taken together, our study found that AR could be involved in the regulation of arachidonic acid metabolism and ether lipid metabolism by regulating the expression of CYP1A2, PCYT1A and CYP1B1, thereby effectively improving liver fibrosis. This study confirmed the advantages of AR in the treatment of liver fibrosis with multiple targets and multiple pathways. In addition, it has been reported that *Asiatic acid* can ameliorate CCl_4_-induced liver fibrosis in rats by regulating PI3K/AKT/mTOR, Bcl-2/Bax, Nrf2/ARE, NF-κB/IκBα and JAK1/STAT3 signaling pathways [47, 48]. *Salvia miltiorrhiza* could inhibit liver fibrosis by regulating TGF-β/Smad, Nrf2/HO-1 and NF-κB/IκBα signaling pathways [49, 50]. These results indicate that multi-channel and multi-target are the advantages of TCM, which has great potential in the treatment of liver fibrosis and deserves further exploration.

## Conclusions

This study confirmed that AR had a therapeutic effect on CCl_4_-induced liver fibrosis, possibly by regulating arachidonic acid metabolism and ether lipid metabolism, which may provide new insights into the mechanism of AR in the treatment of liver fibrosis. In addition, this study also confirmed that TCM has potential advantages in the treatment of liver fibrosis, which is worth further exploring.

## Supplementary information

**Additional file 1: Figure S1**. Q -TOF LC/MS total ion chromatogram in ESI+. **Table S1**. The major chemical compounds of AR extract.

**Additional file 2: Figure S2.** The corresponding loading scores plots from PCA.

**Additional file 3: Table S2.** Differential identified metabolites for discrimination among control and model groups. **Table S3.** Differential identified metabolites for discrimination among model and 10.8 g/kg AR groups.

**Additional file 4: Figure S3.** The OPLS-DA score plots, S-plots and 100-permutation test generated in ESI− mode.

**Additional file 5: Figure S5.** The interrelation between metabolic differences.

## Data Availability

The data used to support the findings of this study are available from the corresponding author upon request.

## Abbreviations

AR: *Astragali Radix*
ALT: alanine transaminase
AST: aspartate transaminase
AKP: alkaline phosphatase
α-SMA: alpha smooth muscle actin
TGF-β1: transforming growth factor-beta 1
PBS: phosphate buffered saline
QC: quality control
UPLC–Q-TOF/MS: ultraperformance liquid chromatography with quadrupole time-of flight mass spectrometry
PCA: principle component analysis
OPLS-DA: orthogonal projection to latent structures discriminate analysis
ESI: electrospray ionization
KEGG: Kyoto Encyclopedia of Genes of Genomes
TCMSP: traditional Chinese medicine systems pharmacology
MOGAT2: 2-acylglycerol *O*-acyltransferase 2
CYP4501A2: cytochrome P4501A2
PCYTIA: choline-phosphate cytidylyltransferase A
CYP3A4: cytochrome P4503A4
CYP4501B1: cytochrome P4501B1
CYP2A6: cytochrome P450 2A6
RT-PCR: real time polymerase chain reaction
CCl_4_: carbon tetrachloride
H&E: hematoxylin–eosin
HMDB: Human Metabolome Database
PPI: protein interaction
TCM: traditional Chinese medicine
