# Supplementary material for: Metabolomics combined with network pharmacology exploration reveals the modulatory properties of Astragali Radix extract in the treatment of liver fibrosis
Source: Chin Med. 2019 Aug 28;14:30. doi: 10.1186/s13020-019-0251-z (PMC6712842; doi:10.1186/s13020-019-0251-z)
Supplement: Supplementary file 1 — Additional file 1: Figure S1. Q -TOF LC/MS total ion chromatogram in ESI+. Table S1. The major chemical compounds of AR extract. [file 13020_2019_251_MOESM1_ESM.docx]

**Materials and Methods**

**1.1. Sample preparation for Q-TOF LC/MS Analysis**

The samples were re-dissolved in water under ultrasound to the concentration of 10 ng/mL, and filtered through 0.22 μm membranes prior to injection.

**1.2. Q-TOF LC/MS Analysis of Chemical Constituents**

The water extract of *Astragali radix*(AR) was analyzed by an Agilent 6550 iFunnel Q-TOF LC/MS (Agilent Technologies, USA). 4 μL of the sample solution was injected and separated by a ZORBOX RRHD C18 analytical column (2.1 mm i.d. × 100 mm, 1.8 μm i.d., Agilent Technologies, USA), eluted using a linear gradient of 0.3 mL/min, as follows: 0-1 min, 100% A; 1-9 min, 100-60% A; 9-19 min, 60-10% A; 19-21 min, 10-0% A; 21-25 min, 100% B (A-0.1% formic acid in water; B-0.1% formic acid in acetonitrile). The eluent was directly introduced into the mass spectrometer. The Agilent 6550 Q-TOF-MS was run in both ESI ^+^ and ESI^-^ modes with capillary voltages of 4000 V and 3500 V respectively. And the mass range was set from *m/z* 50 to 1200. Agilent Mass Hunter Acquisition Software Ver. B.08.00 was used for all operations, acquisitions and data analysis.

**Results**

**Analysis of chemical constituents of AR extract**

In this study, Q-TOF LC/MS technique was used to rapidly analyze and identify the chemical constituents of AR. In the ESI positive mode, the total ion current chromatogram is shown in Supplement Figure 1. The chemical constituents of AR were first separated by HPLC, and then qualitative analysis by the retention time (*t _R_*) values and MS fragment characteristics under positive mode. The results showed that there were 22 major compounds in the extract of AR, which are listed in Additional Table S1.

**Additional Table S1** The major chemical compounds of AR extract.

| No. | *t _R_*  (min) | Formula | Mass | Identification | Area Sum % |
| --- | --- | --- | --- | --- | --- |
| 1 | 1.267 | C_6_H_5_NO_2_ | 123.031 | Nicotinic acid | - |
| 2 | 1.926 | C_9_H_11_NO | 149.085 | D-Cathinone | 2.65 |
| 3 | 4.529 | C_19_H_24_O_6_ | 348.157 | Acantholide | 1 |
| 4 | 4.91 | C_20_H_26_N2O_2_ | 326.194 | Ajmaline | 1.59 |
| 5 | 5.258 | C_22_H_30_N_2_O_3_ | 370.220 | Aspidocarpine | 1.81 |
| 6 | 5.59 | C_8_H_4_O_3_ | 148.016 | Phthalic anhydride | 1.76 |
| 7 | 5.822 | C_51_H_82_O_22_ | 1046.53 | (24S,25R) -24-Hydroxyspirost-5-en-3beta-yl O-alpha-L-rhamnopyranosyl-(1-->2) -O-[O-beta-D-glucopyran | 1.38 |
| 8 | 6.44 | C_22_H_22_O_10_ | 446.12 | Calycosin-7-glucoside | 0.69 |
| 9 | 7.032 | C_39_H_66_O_14_ | 758.443 | Anemarrhenasaponin-I | 4.55 |
| 10 | 7.331 | C_26_H_34_O_10_ | 506.214 | Massonianoside C | 3.24 |
| 11 | 7.563 | C_51_H_86_O_23_ | 1066.55 | Asparanin B8 | 1.38 |
| 12 | 8.16 | C_22_H_22_O_9_ | 430.125 | Formononetin-7-glucoside | - |
| 13 | 9.171 | C_16_H_12_O_5_ | 284.069 | Calycosin | 2 |
| 14 | 10.016 | C_20_H_30_O_5_ | 350.206 | Andrographolide | 2.57 |
| 15 | 10.547 | C_41_H_68_O_14_ | 807.50 | Astragaloside IV | - |
| 16 | 11.459 | C_16_H_12_O_4_ | 268.074 | Formononetin | 15.4 |
| 17 | 12.304 | C_43_H_70_O_15_ | 826.469 | Astragaloside II | - |
| 18 | 13.149 | C_14_H_18_O_2_ | 218.13 | Pterosin B | 7.46 |
| 19 | 16.117 | C_24_H_30_O_6_ | 414.203 | Armillaripin | 10.31 |
| 20 | 19.764 | C_22_H_36_O_8_ | 428.241 | Rhodomollein III | 7.05 |
| 21 | 21.041 | C_7_H_6_O_3_ | 138.032 | 3,4-Dihydroxybenzyl aldehyde | 6.17 |
| 22 | 21.107 | C_16_H_25_NO_2_ | 263.183 | Clavatine | 6.46 |


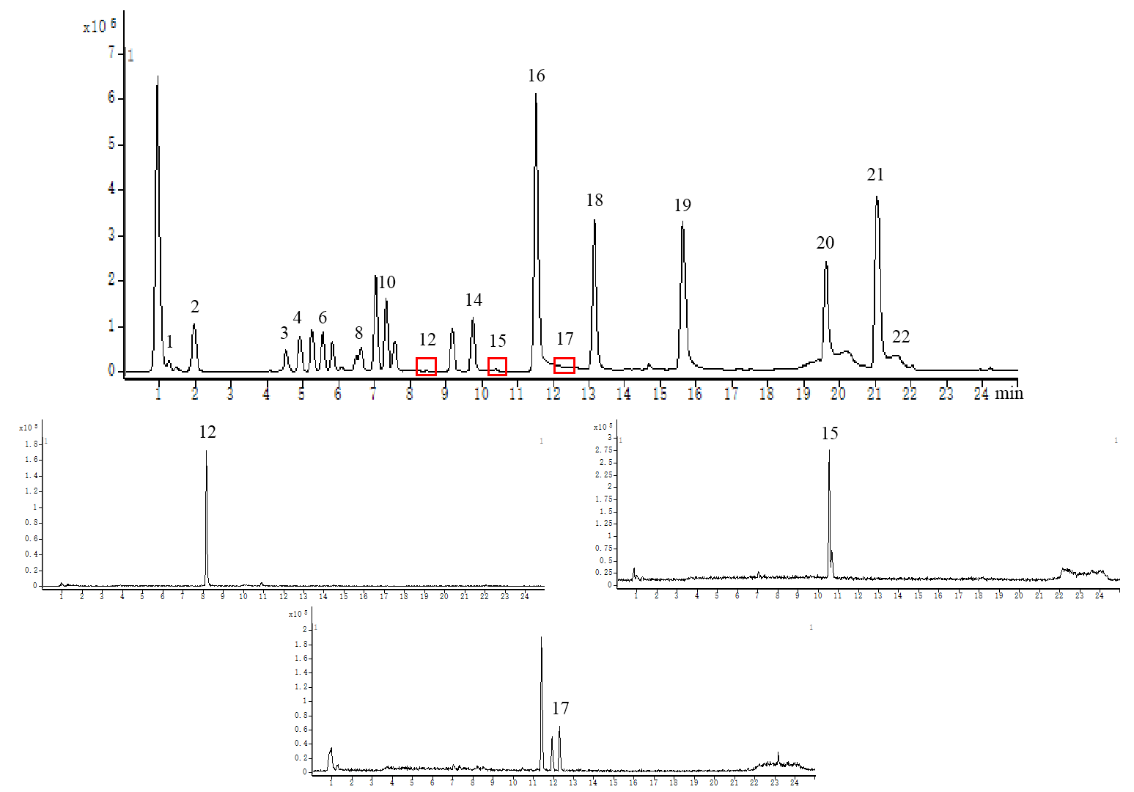


**Additional Figure S1** Q -TOF LC/MS total ion chromatogram in ESI^+^.
