# Supplementary figures and images for: Metabolomics combined with network pharmacology exploration reveals the modulatory properties of Astragali Radix extract in the treatment of liver fibrosis
Source: Chin Med. 2019 Aug 28;14:30. doi: 10.1186/s13020-019-0251-z (PMC6712842; doi:10.1186/s13020-019-0251-z)

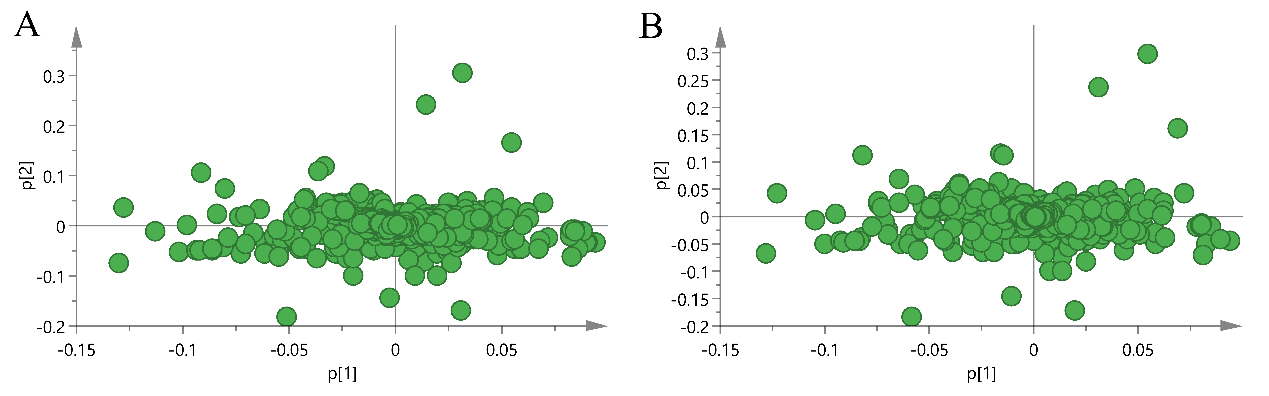


**Additional Figure S2.** The corresponding loading scores plots from PCA. (A) ESI+ mode. (B) ESI- mode.

Supplement: Supplementary file 2 — Additional file 2: Figure S2. The corresponding loading scores plots from PCA. [file 13020_2019_251_MOESM2_ESM.docx]
