# Supplementary material for: Metabolomics combined with network pharmacology exploration reveals the modulatory properties of Astragali Radix extract in the treatment of liver fibrosis
Source: Chin Med. 2019 Aug 28;14:30. doi: 10.1186/s13020-019-0251-z (PMC6712842; doi:10.1186/s13020-019-0251-z)
Supplement: Supplementary file 3 — Additional file 3: Table S2. Differential identified metabolites for discrimination among control and model groups. Table S3. Differential identified metabolites for discrimination among model and 10.8 g/kg AR groups. [file 13020_2019_251_MOESM3_ESM.docx]

**Additional Table S2** Differential identified metabolites for discrimination among control and model groups.

| No. | R.T. (min) | Mass (*m/z*) | Metabolites |
| --- | --- | --- | --- |
| ESI+ | | | |
| 1 | 17.067 | 546.3547 | LysoPC(20:3(5Z,8Z,11Z)) |
| 2 | 16.987 | 524.3710 | 2-Acetyl-1-alkyl-sn-glycero-3-phosphocholine |
| 3 | 21.080 | 124.0868 | 4-Hydroxybenzylamine |
| 4 | 20.235 | 124.0868 | 1-Ethyl-1H-pyrrole-2-carboxaldehyde |
| 5 | 16.392 | 421.2336 | Levocabastine |
| 6 | 20.174 | 828.5534 | PC(22:6(4Z,7Z,10Z,13Z,16Z,19Z)/18:3(9Z,12Z,15Z)) |
| 7 | 16.404 | 422.2360 | PC-M6 |
| 8 | 16.363 | 343.2239 | 5,6-Epoxy-8,11,14-eicosatrienoic acid |
| 9 | 16.387 | 281.2664 | Linoleic acid |
| 10 | 22.070 | 899.7163 | TG(18:3(6Z,9Z,12Z)/20:4(5Z,8Z,11Z,14Z)/18:3(6Z,9Z,12Z)) |
| 11 | 11.100 | 319.2284 | Leukotriene A4 |
| 12 | 12.890 | 355.2825 | MG(0:0/18:2(9Z,12Z)/0:0) |
| 13 | 21.950 | 900.7125 | PC(20:0/24:1(15Z)) |
| 14 | 22.159 | 894.7360 | PC(20:3(5Z,8Z,11Z)/24:1(15Z)) |
| 15 | 17.598 | 726.5365 | PE(16:0/P-18:0) |
| 16 | 18.150 | 322.2696 | 3-Dehydrosphinganine |
| 17 | 10.064 | 371.2354 | 6-Keto-prostaglandin F1a |
| 18 | 18.113 | 322.2696 | Sphingosine |
| 19 | 22.022 | 894.7292 | PC(20:3(8Z,11Z,14Z)/24:1(15Z)) |
| 20 | 14.487 | 1087.6831 | CL(8:0/10:0/11:0/i-16:0) |
| 21 | 19.155 | 759.5731 | PA(16:0/24:1(15Z)) |
| 22 | 14.781 | 522.3435 | 2-oleoyl-sn-glycero-3-phosphocholine |
| 23 | 21.675 | 124.0868 | 2-Acetyl-1-methylpyrrole |
| 24 | 19.694 | 828.5510 | PC(22:6(4Z,7Z,10Z,13Z,16Z,19Z)/16:0) |
| 25 | 16.030 | 532.3398 | LysoPC(O-18:0) |
| 26 | 11.471 | 355.2418 | 9,10,13-Trihydroxystearic acid |
| 27 | 14.224 | 544.3388 | LysoPC(18:1(9Z)) |
| 28 | 17.080 | 547.3592 | 1-Stearoylglycerophosphocholine |
| 29 | 12.021 | 373.2470 | Neriantogenin |
| ESI- | | | |
| 30 | 16.392 | 421.2336 | 8-Hydroxycarvedilol |
| 31 | 7.792 | 273.1672 | 3-Hydroxytetradecanedioic acid |
| 32 | 16.404 | 422.2360 | LysoPE(0:0/14:1(9Z)) |
| 33 | 14.174 | 624.2813 | Leukotriene C4 |
| 34 | 16.387 | 281.2664 | Ethyl 9-hexadecenoate |
| 35 | 11.100 | 319.2284 | 5,6-Epoxy-8,11,14-eicosatrienoic acid |
| 36 | 15.418 | 391.2163 | Dihydrofukinolide |
| 37 | 14.214 | 377.2692 | MG(0:0/20:4(8Z,11Z,14Z,17Z)/0:0) |
| 38 | 13.390 | 377.2677 | 2-Arachidonylglycerol |
| 39 | 15.704 | 365.2065 | 20-Carboxy-leukotriene B4 |
| 40 | 22.166 | 895.7307 | TG(15:0/22:2(13Z,16Z)/18:2(9Z,12Z)) |
| 41 | 12.879 | 494.3213 | LysoPC(16:0) |
| 42 | 20.192 | 781.5566 | PA(22:2(13Z,16Z)/20:1(11Z)) |
| 43 | 14.832 | 590.3120 | Nummularine B |
| 44 | 16.599 | 504.3138 | LysoPE(0:0/20:2(11Z,14Z)) |
| 45 | 14.609 | 498.3348 | Taurochenodesoxycholic acid |
| 46 | 19.709 | 828.5510 | PC(22:6(4Z,7Z,10Z,13Z,16Z,19Z)/18:2(9Z,12Z)) |
| 47 | 16.618 | 504.3121 | LysoPE(20:2(11Z,14Z)/0:0) |
| 48 | 19.795 | 781.5542 | PA(20:3(8Z,11Z,14Z)/22:0) |
| 49 | 16.518 | 301.1600 | Glutaminylarginine |

**Additional Table S3** Differential identified metabolites for discrimination among model and 10.8g/kg AR groups.

| No. | R.T. (min) | Mass (*m/z*) | Metabolites |
| --- | --- | --- | --- |
| ESI+ | | | |
| 1 | 18.676 | 811.6064 | PA(22:0/20:0) |
| 2 | 16.987 | 524.3710 | 2-Acetyl-1-alkyl-sn-glycero-3-phosphocholine |
| 3 | 20.176 | 282.2792 | Oleamide |
| 4 | 17.478 | 352.2511 | 4,8 Dimethylnonanoyl carnitine |
| 5 | 19.432 | 836.6131 | PC(16:0/22:2(13Z,16Z)) |
| 6 | 22.318 | 918.7295 | PC(22:5(4Z,7Z,10Z,13Z,16Z)/24:1(15Z)) |
| 7 | 22.357 | 917.7191 | TG(20:2n6/15:0/20:3n6) |
| 8 | 22.428 | 429.3392 | 3,5-Dihydroxyergosta-7,22-dien-6-one |
| 9 | 16.363 | 343.2239 | 5,6-Epoxy-8,11,14-eicosatrienoic acid |
| 10 | 19.227 | 814.6778 | PE(P-18:1(9Z)/24:0) |
| 11 | 13.420 | 438.2013 | N1,N10-Dicoumaroylspermidine |
| 12 | 13.368 | 460.2684 | PE(P-16:0e/0:0) |
| 13 | 21.681 | 901.7125 | TG(18:2(9Z,12Z)/18:2(9Z,12Z)/18:2(9Z,12Z)) |
| 14 | 21.479 | 899.7155 | TG(18:3(9Z,12Z,15Z)/20:4(5Z,8Z,11Z,14Z)/18:3(9Z,12Z,15Z)) |
| 15 | 18.808 | 734.5690 | PC(16:0/16:0) |
| 16 | 16.282 | 482.3341 | LysoPC(15:0) |
| 17 | 19.106 | 760.5824 | PC(14:0/20:1(11Z)) |
| 18 | 18.150 | 322.2696 | 3-Dehydrosphinganine |
| 19 | 10.064 | 371.2354 | 6-Keto-prostaglandin F1a |
| 20 | 18.113 | 322.2696 | Sphingosine |
| 21 | 19.468 | 836.6152 | PE-NMe(16:1(9Z)/24:1(15Z)) |
| 22 | 20.006 | 734.5667 | PC(18:0/14:0) |
| 23 | 21.733 | 895.7281 | PA(24:0/24:0) |
| 24 | 14.125 | 568.3403 | LysoPC(22:6(4Z,7Z,10Z,13Z,16Z,19Z)) |
| 25 | 19.309 | 814.6684 | PE(P-18:0/24:1(15Z)) |
| 26 | 16.599 | 504.3138 | LysoPE(0:0/20:3(5Z,8Z,11Z)) |
| 27 | 18.205 | 783.5638 | PA(a-21:0/i-19:0) |
| 28 | 17.625 | 352.2529 | Sphingosine 1-phosphate (d16:1-P) |
| 29 | 16.030 | 532.3398 | LysoPC(O-18:0) |
| 30 | 18.246 | 280.2636 | Linoleamide |
| 31 | 14.224 | 544.3388 | LysoPC(18:1(9Z)) |
| 32 | 21.853 | 894.7174 | PC(20:4(8Z,11Z,14Z,17Z)/24:0) |
| ESI- | | | |
| 33 | 14.781 | 522.3444 | 2-acetyl-1-alkyl-sn-glycero-3-phosphocholine |
| 34 | 22.008 | 899.7164 | TG(18:3(6Z,9Z,12Z)/16:1(9Z)/22:5(7Z,10Z,13Z,16Z,19Z)) |
| 35 | 13.719 | 339.2998 | 6-Hydroxy-8-docosanone |
| 36 | 19.872 | 304.2609 | Arginyl-Methionine |
| 37 | 17.605 | 352.2520 | PE(24:0/P-18:0) |
| 38 | 19.468 | 813.6614 | PA(20:1(11Z)/24:0) |
| 39 | 19.432 | 836.6131 | PC(20:3(8Z,11Z,14Z)/20:1(11Z)) |
| 40 | 14.174 | 624.2813 | Leukotriene C4 |
| 41 | 20.252 | 918.5625 | PS(22:5(4Z,7Z,10Z,13Z,16Z)/24:1(15Z)) |
| 42 | 10.951 | 431.2362 | Glucosyl (2E,6E,10x)-10,11-dihydroxy-2,6-farnesadienoate |
| 43 | 22.159 | 896.7393 | PE-NMe(24:0/22:2(13Z,16Z)) |
| 44 | 19.227 | 814.6778 | PE(P-18:0/24:0) |
| 45 | 22.264 | 896.7410 | PE-NMe(22:2(13Z,16Z)/24:0) |
| 46 | 18.297 | 517.3708 | Ganoderiol C |
| 47 | 21.681 | 901.7125 | TG(18:4(6Z,9Z,12Z,15Z)/18:0/20:4(8Z,11Z,14Z,17Z)) |
| 48 | 22.231 | 915.7285 | TG(20:3(5Z,8Z,11Z)/15:0/22:5(4Z,7Z,10Z,13Z,16Z)) |
| 49 | 22.365 | 918.7300 | PC(22:4(7Z,10Z,13Z,16Z)/24:1(15Z)) |
| 50 | 18.809 | 734.5690 | PS(16:0/16:0) |
| 51 | 14.203 | 568.3368 | LysoPC(22:5(4Z,7Z,10Z,13Z,16Z)) |
| 52 | 14.936 | 570.3555 | LysoPC(22:4(7Z,10Z,13Z,16Z)) |
| 53 | 22.189 | 915.7267 | TG(15:0/22:5(7Z,10Z,13Z,16Z,19Z)/20:3n6) |
| 54 | 14.609 | 498.3348 | Taurochenodesoxycholic acid |
| 55 | 13.411 | 460.2698 | Glucosylsphingosine |
| 56 | 22.686 | 935.7348 | TG(22:6(4Z,7Z,10Z,13Z,16Z,19Z)/15:0/22:6(4Z,7Z,10Z,13Z,16Z,19Z)) |
| 57 | 18.246 | 280.2636 | 8-hydroxymirtazapine |
