# Supplementary material for: Metabolomics combined with network pharmacology exploration reveals the modulatory properties of Astragali Radix extract in the treatment of liver fibrosis
Source: Chin Med. 2019 Aug 28;14:30. doi: 10.1186/s13020-019-0251-z (PMC6712842; doi:10.1186/s13020-019-0251-z)
Supplement: Supplementary file 4 — Additional file 4: Figure S3. The OPLS-DA score plots, S-plots and 100-permutation test generated in ESI− mode. [file 13020_2019_251_MOESM4_ESM.docx]

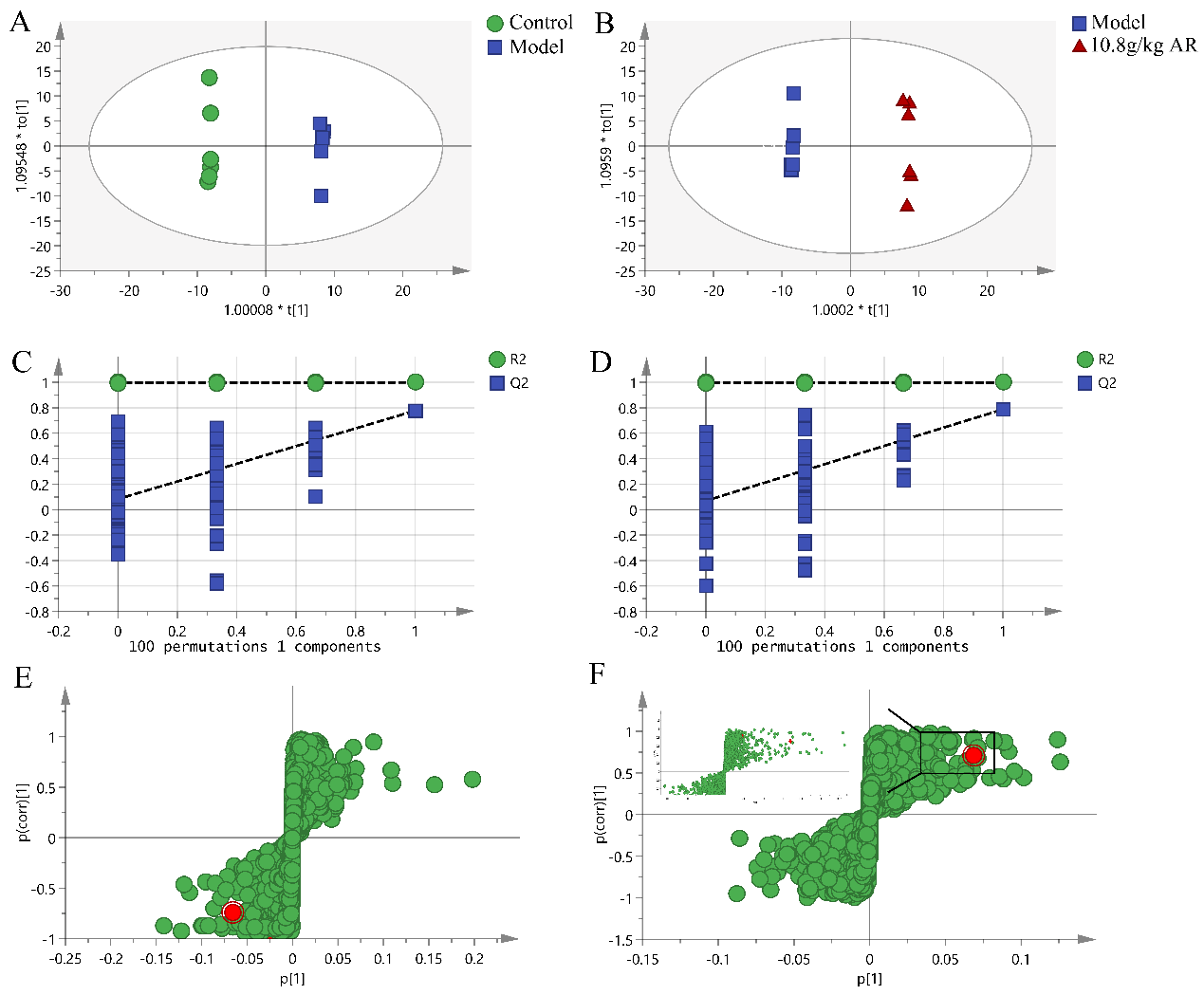


**Additional Figure S3.** The OPLS-DA score plots, S-plots and 100-permutation test generated in ESI- mode. OPLS-DA score plots were the pair-wise comparisons between the control and model groups (A) as well as between the model and AR groups (B). The 100-permutation test of the OPLS-DA model was for the control and model groups (C) as well as for the model and AR groups (D). S-plots of the OPLS-DA model for the control and model groups (E) as well as for the model and AR groups (F).
