# Supplementary material for: Metabolomics combined with network pharmacology exploration reveals the modulatory properties of Astragali Radix extract in the treatment of liver fibrosis
Source: Chin Med. 2019 Aug 28;14:30. doi: 10.1186/s13020-019-0251-z (PMC6712842; doi:10.1186/s13020-019-0251-z)
Supplement: Supplementary file 5 — Additional file 5: Figure S5. The interrelation between metabolic differences. [file 13020_2019_251_MOESM5_ESM.docx]

**
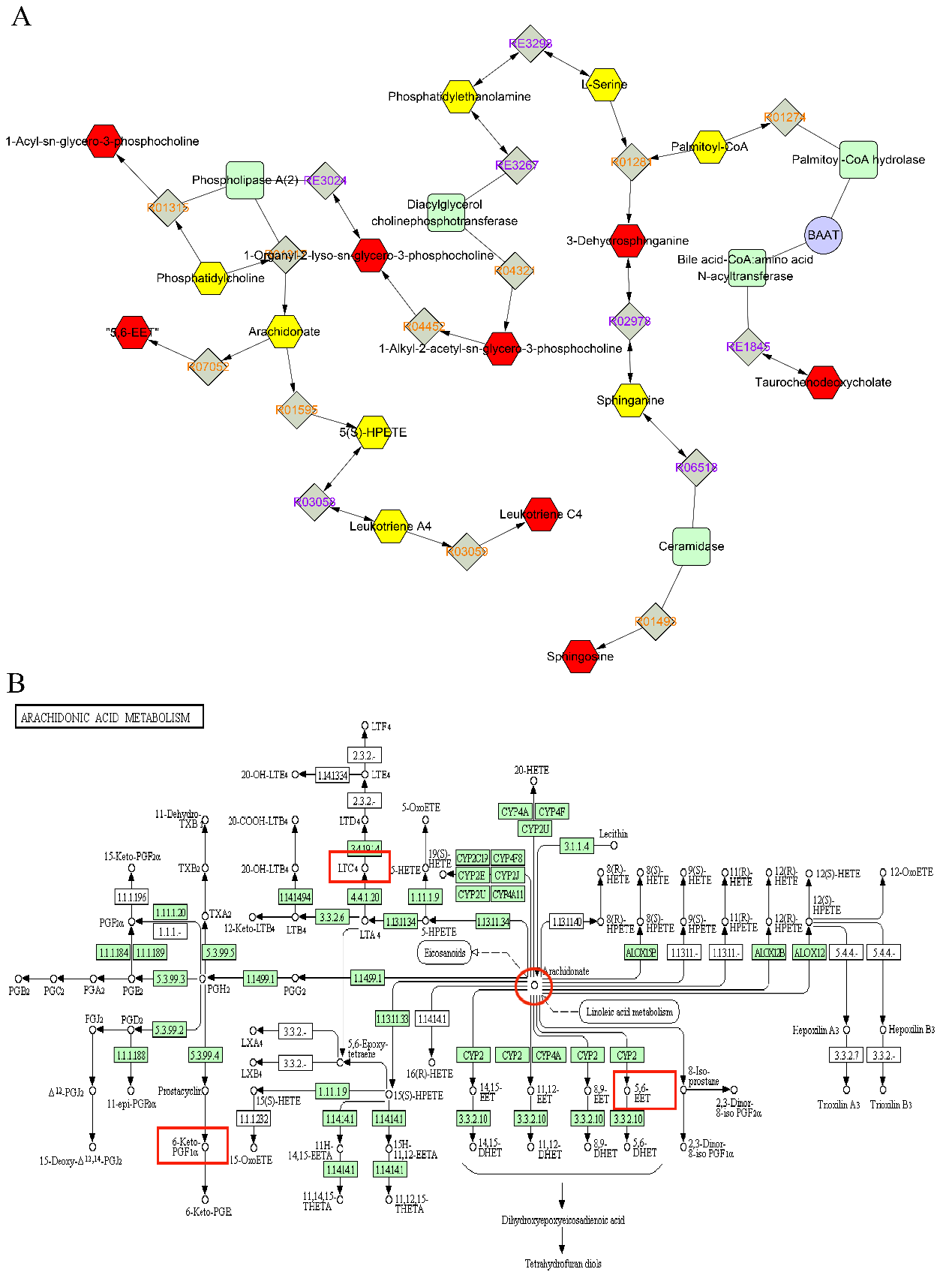
**

**Additional Figure S4.** The interrelation between metabolic differences. (A) MetScape was used to analyze 8 metabolites. (B) KEGG pathway was used to analyze differential metabolites. The hexagon represents the compound node, in which the red hexagon represents the metabolic difference we found. The diamond represents the reaction node, and the square represents the enzyme node.
